# Hippocampal snRNA‐seq in Collaborative Cross reveals molecular signatures of cognitive resilience independent of chronological aging

**DOI:** 10.1002/alz.71825

**Published:** 2026-09-16

**Authors:** Yu Chen, Andrew R. Ouellette, Yiding Cao, Jigang Zhang, Catherine C. Kaczorowski

**Affiliations:** ^1^ Department of Neurology University of Michigan Ann Arbor Michigan USA; ^2^ The Jackson Laboratory Bar Harbor Maine USA; ^3^ University of Louisville Louisville Kentucky USA

**Keywords:** cognitive resilience, Collaborative Cross, dementia, genetically diverse mouse reference panel, glutathione metabolism, proteostasis, snRNA‐seq

## Abstract

**INTRODUCTION:**

Genetic factors contribute to variability in age‐related cognitive decline, yet the molecular mechanisms underlying cognitive resilience remain poorly understood.

**METHODS:**

We performed single‐nucleus RNA sequencing on hippocampal tissue from 107 genetically diverse Collaborative Cross mice across three ages (6, 12, and 18 months) and both sexes. Contextual fear memory was used as a continuous measure of cognitive function. Pseudobulk differential expression analyses identified transcriptional changes associated with aging and cognitive decline.

**RESULTS:**

Aging and cognitive decline exhibited differential transcriptional signatures. Aging‐related changes were modest and cell‐type specific, with early responses in excitatory neurons followed by increased glial remodeling. Conversely, cognitive decline was associated with widespread transcriptional alterations predominantly in excitatory neurons, enriched for proteostasis and glutathione/redox pathways.

**DISCUSSION:**

These findings indicate that cognitive decline is not simply a consequence of aging but reflects distinct molecular processes in excitatory neurons. Proteostasis and redox pathways may represent conserved mechanisms supporting cognitive resilience.

## Highlights

- Aging and cognitive decline show largely separate hippocampal transcriptional signatures.
- Cognitive decline signatures are concentrated in excitatory neurons.
- Aging shifts from early neuronal to later glial transcriptional remodeling.
- Proteostasis and glutathione pathways are linked to cognitive resilience.
- Genetic diversity reveals molecular mechanisms of resilient cognitive aging.

## 1 BACKGROUND

Although Alzheimer's disease (AD) is the most prevalent form of dementia, aging itself remains one of the most prominent risk factors for cognitive decline and dementia.^1^ However, the trajectory of cognitive aging varies substantially across individuals. While some individuals maintain relatively normal cognitive function well into late life, others develop measurable impairments much earlier than expected.^2^, ^3^ At the extreme end of this spectrum are so‐called Super Agers, individuals who exhibit superior cognitive performance compared to their age‐matched peers.^4^, ^5^, ^6^ This variability highlights an important distinction between chronological aging and cognitive aging. Cases of preserved cognition have also been reported in pathology‐related dementias, where individuals maintain normal cognitive function despite significant neuropathological burden.^7^, ^8^ Indeed, accumulating evidence suggests that chronological aging does not necessarily coincide with cognitive decline.^9^ These observations indicate that cognitive decline is not an inevitable consequence of aging and that molecular mechanisms exist that promote resilience to age‐related cognitive impairment.

Genetic variation is thought to play a major role in shaping this variability. Studies suggest that up to 60% to 70% of the variation in cognitive abilities during aging can be attributed to genetic influences,^10^ although the precise variants and mechanisms involved remain difficult to identify. Therefore, understanding the molecular pathways that contribute to cognitive resilience represents a major challenge in aging biology and has important implications for developing therapeutic strategies to preserve cognitive health.

Animal models play a critical role in elucidating the molecular and cellular mechanisms underlying cognitive aging and resilience, as they allow invasive interrogation of molecular and cellular changes across multiple time points, whereas studies in humans are largely limited to end‐point measurements.^3^ However, conventional mouse models are typically maintained on a single genetic background, most commonly C57BL/6J (B6), which fails to capture the genetic diversity that influences cognitive decline. A recent study across multiple neurodegenerative diseases, including AD, frontotemporal dementia (FTD), and progressive supranuclear palsy (PSP), demonstrate that the genetic architectures of these disorders are distinct and involve differential contributions of neurons and glia to disease risk.^11^ These findings suggest that genetic background influences which molecular mechanisms are engaged during neurodegeneration and cognitive decline, a dimension that single‐background models cannot capture.

Genetically diverse mouse populations offer an important alternative for studying complex traits influenced by genetic background. The Collaborative Cross (CC) is a recombinant inbred mouse population derived from eight founder strains and designed to capture high levels of genetic diversity.^12^ Previous studies across multiple biological fields successfully leveraged the CC population to dissect the genetic architecture of complex traits, including immune responses,^13^, ^14^ infectious disease susceptibility,^15^, ^16^ metabolic phenotypes,^17^, ^18^ neurodegenerative diseases,^19^ and neurological traits.^20^, ^21^ These studies highlight the power of genetically diverse populations to identify genetic modifiers that are often undetectable in traditional inbred mouse models.

In this study, we leveraged the genetic diversity of the CC population to examine how chronological aging and cognitive decline are reflected at the molecular level in the hippocampus. Specifically, we examined whether transcriptional changes associated with cognitive decline simply mirrored those associated with aging or whether they represented molecularly separable processes. The CC population is well suited to answer these questions as it captures a broad range of genetic backgrounds and cognitive aging trajectories while allowing molecular profiling under controlled experimental conditions. By combining cognitive performance testing with hippocampal single‐nucleus RNA sequencing (snRNA‐seq) across multiple biological variables including age, sex, and strain, this study highlights cell‐type‐specific transcriptional signatures associated with cognitive aging and resilience.

## 2 METHODS

### 2.1 Animals

All animal procedures were performed in accordance with the standards of the Association for the Assessment and Accreditation of Laboratory Animal Care and the National Institutes of Health (NIH) Guide for the Care and Use of Laboratory Animals and were approved by the respective Institutional Animal Care and Use Committees. Mice were group‐housed at The Jackson Laboratory under a standard 12:12 light‐dark cycle (lights on at 0600 h) with ad libitum access to NIH31 5K52 chow (LabDiet/PMI Nutrition, St. Louis, MO, USA) and acidified water. Mice were housed in duplex polycarbonate cages on ventilated racks providing 99.997% HEPA‐filtered air in a climate‐controlled room, with pine bedding changed weekly. Singly housed mice were provided enrichment in the form of a Shepherd Shack. All experiments were performed during the light phase of the 12‐h light‐dark cycle.

Eight CC strains were used in this study: CC003, CC004, CC005, CC006, CC019, CC032, CC041, and CC061. CC mice were obtained from The Jackson Laboratory via the Nathan Shock Center of Excellence in the Basic Biology of Aging as part of a cross‐sectional phenotyping project in which independent cohorts of male and female mice were timed for nearly simultaneous testing at 6, 12, and 18 months of age to avoid repeated testing. A total of 340 mice were included in the overall study cohort (Table S1).

### 2.2 Behavioral data and tissue collection

All mice underwent a series of behavioral phenotyping assessments including Y‐maze, inclined screen, grip strength, and contextual fear memory (CFM). Detailed protocols for these tasks have been described previously.^22^, ^23^, ^24^ Among these, CFM was used as the primary cognitive outcome in this study. Briefly, FreezeFrame software (Coulbourn Instruments, Whitehall Township, PA, USA) was utilized for behavioral assessment. The CFC protocol consisted of a 180‐s baseline period followed by four mild foot shocks (1 s, 0.9 mA), each separated by intervals of 115 ± 20 s. After each shock, a 40‐s post‐shock (PS) interval was established, and the percentage of time spent freezing during each of these intervals was recorded. The percent time freezing during the fourth PS interval (PS4) served as an index of contextual fear acquisition (CFA). Twenty‐four hours after training, mice were returned to the conditioning chamber for a 10‐min testing period, during which the percent time freezing was recorded as an index of CFM. CFM testing was performed terminally prior to tissue harvest. Upon completion of behavioral testing, hippocampal tissue was dissected and snap‐frozen in liquid nitrogen. RNA was extracted using the RNeasy kit with the QIAcube (Qiagen) according to the manufacturer's protocol. Samples were subsequently sent to the JAX Advanced Precision Medicine Laboratory for processing and shipped on dry ice.

### 2.3 Single‐nucleus RNA sequencing

SnRNA‐seq libraries were prepared and sequenced by the JAX Advanced Precision Medicine Laboratory, and sequencing reads were aligned to the mouse mm10 pre‐mRNA reference genome to generate raw count matrices. Nuclei with greater than 5% mitochondrial reads were excluded from downstream analysis. Raw count matrices were processed in R (version 3.6.0) using Seurat (version 3.1.1).^25^ Data were normalized using the NormalizeData function, and variable features were identified using the variance‐stabilizing transformation (VST) method. Data were scaled with regression of sequencing depth (nCount_RNA) and proportions of ribosomal protein subunit genes and pseudogene‐mapped reads. Principal component analysis (PCA) was performed, and the number of variable features and principal components were optimized by systematically evaluating clustering outcomes across a range of parameters; the final analysis used 2000 variable features and 25 principal components.

Batch correction for sequencing run and date of tissue preparation was performed using Harmony (version 1.0).^26^ Shared nearest‐neighbor (SNN) graph construction and graph‐based clustering were carried out using the Harmony‐corrected embeddings across a range of resolutions, and a final resolution of 0.3 was selected. Uniform Manifold Approximation and Projection (UMAP) representations were generated for visualization.

**RESEARCH IN CONTEXT**

1. **Systematic review**: Although aging is the prominent risk factor for cognitive decline and dementia, chronological aging and cognitive decline do not inevitably coincide. Genetic factors are thought to play a major role in this variability, yet the molecular pathways conferring resilience to age‐related cognitive impairment remain poorly understood.
2. **Interpretation**: Using hippocampal snRNA‐seq in genetically diverse CC mice, we found that chronological aging and cognitive decline exhibited largely distinct transcriptional signatures. Aging produced relatively modest, cell‐type‐specific changes, whereas cognitive decline was associated with extensive transcriptional remodeling concentrated in excitatory neurons and enriched for proteostasis, translation, and glutathione/redox pathways.
3. **Future directions**: Future studies will further validate the genetic regulators of these resilience‐associated pathways and determine whether modulation of proteostasis and redox homeostasis can preserve cognitive function during aging.

Doublet detection was performed per sample using DoubletFinder (version 2.0.2),^27^ assuming an expected doublet rate of 5%. The optimal neighborhood parameter (pK) was identified empirically via parameter sweep, and homotypic doublet proportions were estimated from cluster‐level annotations to adjust expected doublet counts accordingly. Clusters in which 30% or more of nuclei were classified as doublets were removed in their entirety, and the remaining doublet‐labeled nuclei were subsequently excluded prior to downstream analyses.

Following quality control and doublet removal, initial clustering at resolution 0.3 yielded 20 transcriptionally distinct clusters prior to cell‐type annotation (Figure S1). Quality control metrics, including detected gene counts, RNA counts, mitochondrial read percentage, and pseudogene‐mapped read percentage, were within acceptable ranges across all clusters, with mitochondrial content below 5% (Figure S2). The number of RNA counts and features were strongly correlated (*r* = 0.96), while mitochondrial content showed the expected negative correlation with sequencing depth (*r *= −0.48), confirming overall library quality (Figure S3).

### 2.4 Differential genes expression analysis

Pseudobulk differential expression analysis was performed separately for each major hippocampal cell type and subsequently for each excitatory neuron subtype using DESeq2 (version 1.48.2).^28^ For each cell type, snRNA counts were aggregated by sample using the AggregateExpression() function in Seurat, which sums counts across cells within each group by default, thereby generating pseudobulk count matrices. Sample‐level metadata including age, sex, CC strain, and strain‐specific cognitive decline slope were merged to the corresponding pseudobulk profiles prior to analysis.

Two differential expression analyses were performed. First, to characterize transcriptional changes associated with chronological aging, pairwise comparisons were performed between age groups (12 vs 6 months, 18 vs 6 months, and 18 vs 12 months), with age included as the primary variable and sex as covariate in the DESeq2 design formula. Second, cognitive trajectory was quantified separately for each strain by fitting CFM performance as a function of age, with sex included as a covariate. The age coefficient represented the strain‐specific rate of cognitive change. Because independent cohorts were tested at 6, 12, and 18 months, this coefficient estimates a cross‐sectional strain‐level trajectory rather than longitudinal change within individual animals. Cognitive resilience was defined as relative preservation of CFM across age: a more negative slope indicated steeper age‐associated decline, whereas a slope closer to zero or above zero indicated greater preservation and, therefore, greater resilience. This slope was z‐scored across strains and used as a continuous predictor in the DESeq2 design. We retained this continuous measure to avoid an arbitrary dichotomization of strains in order to preserve information from intermediate trajectories and maintain statistical power. Similar continuous‐trait approaches have been used to quantify cognitive resilience in genetically diverse AD‐BXD populations.^24^ For chronological aging, genes with false discovery rate (FDR) < 0.05 and |log2 fold change| > 0.25 were considered significant. For cognitive trajectory, genes with FDR < 0.05 and |β| > 0.25 were considered significant; positive coefficients were interpreted as associations with more preserved cognitive trajectories, whereas negative coefficients were interpreted as associations with steeper cognitive decline. All FDR corrections used the Benjamini‐Hochberg procedure, and genes with undefined FDR values were excluded.

### 2.5 Functional enrichment and protein‐protein interaction (PPI) network analysis

Gene set enrichment analysis (GSEA) was performed separately for each cell type using clusterProfiler::gseGO() with Gene Ontology Biological Process terms and mouse annotations from org.Mm.eg.db.^29^ Genes were ranked by log2 fold change after removing entries with missing values and duplicate gene symbols, and cell types with fewer than 50 genes were excluded. Enrichment was conducted using gene symbols (keyType = “SYMBOL”) with gene set sizes between 10 and 500 genes and a *p* value cutoff of 0.05. Redundant GO terms were reduced using simplify with the Wang semantic similarity measure (cutoff = 0.7), retaining representative terms based on the minimum adjusted *p* value.

Functional enrichment analysis for differentially expressed genes (DEGs) was separated into up‐ and downregulated sets and analyzed independently using Metascape.^30^ For each gene list, pathway and process enrichment was assessed across multiple annotation sources, including GO Biological Processes, Kyoto Encyclopedia of Genes and Genomes, Reactome, CORUM, WikiPathways, and PANTHER. Enrichment significance was evaluated using a cumulative hypergeometric test with all genes in the genome as the background, and *p* values were adjusted using the Benjamini–Hochberg method. Terms with adjusted *p* value < 0.01, gene count ≥ 3, and enrichment factor > 1.5 were retained. Enriched terms were simplified based on semantic similarity using the Wang method,^31^ and redundant terms were removed using a similarity cutoff of 0.7.

PPI analysis was assessed across multiple sources, including STRING, BioGrid, OmniPath, and InWeb_IM, considering only physical interactions in STRING (physical score > 0.132). Networks containing three to 500 proteins were further analyzed using the MCODE algorithm to identify densely connected modules.^32^ Each module was then subjected to enrichment analysis, and the top three terms ranked by FDR were used to annotate the modules.

## 3 RESULTS

### 3.1 Hippocampal snRNA‐seq across genetically diverse aging mice identifies major cell types with composition differences driven by genetic background and age

**FIGURE 1:**
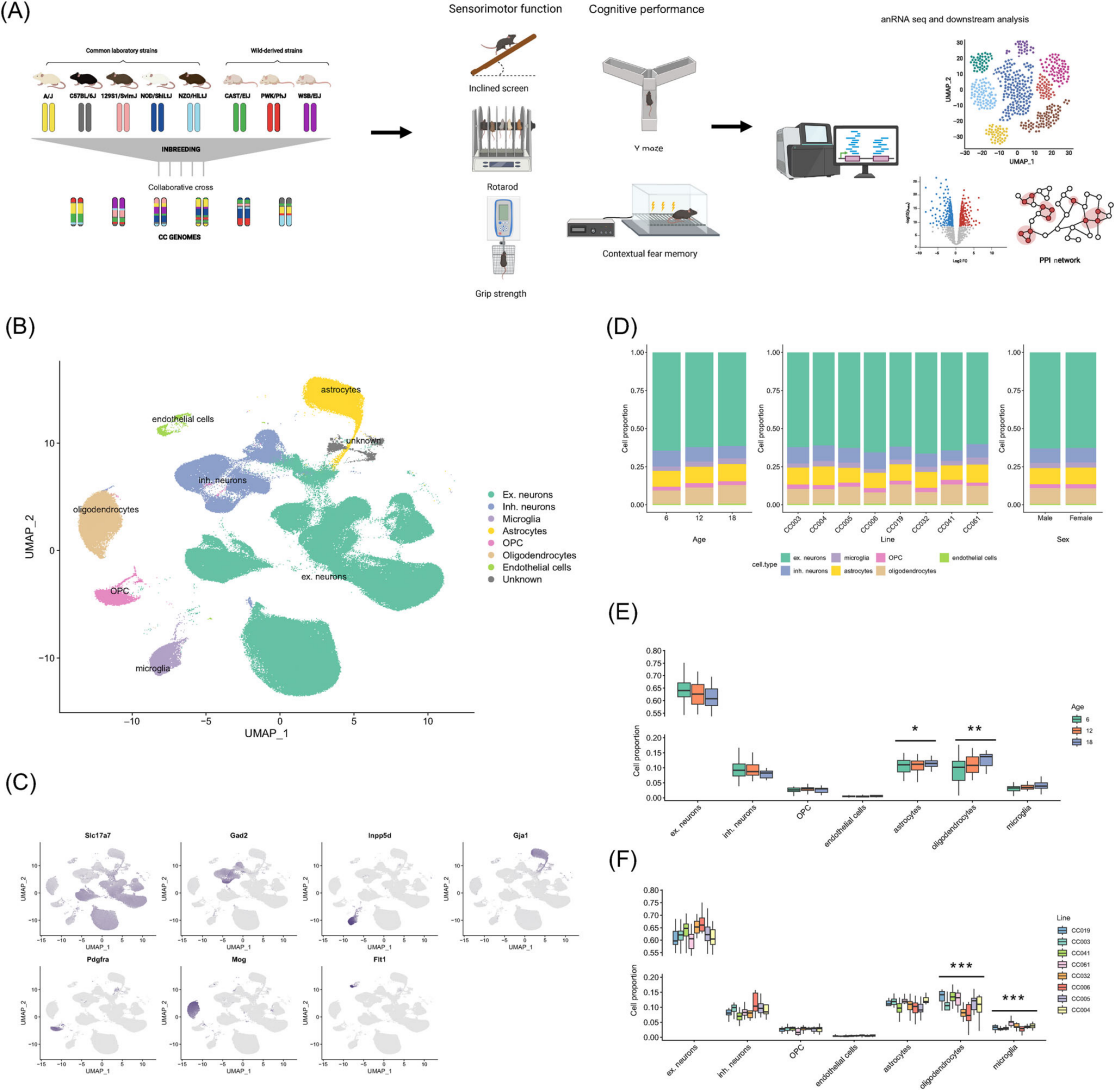
snRNA‐seq sequencing of the hippocampus across CC strains identifies major cell types and reveals composition differences associated with genetic background and age. (A) Schematic overview of study design. CC strains were analyzed across three age points (6, 12, and 18 months) and both sexes. All animals underwent a behavioral assessment battery including rotarod, grip strength, Y‐maze, and contextual fear memory prior to hippocampal tissue collection for snRNA‐seq and downstream analysis. (B) UMAP visualization of eight major cell populations: excitatory neurons, inhibitory neurons, microglia, astrocytes, OPCs, oligodendrocytes, endothelial cells, and an unknown cluster. (C) Expression of canonical cell type marker genes projected onto UMAP space, including Slc17a7 (excitatory neurons), Gad2 (inhibitory neurons), Inpp5d (microglia), Gja1 (astrocytes), Pdgfra (OPCs), Mog (oligodendrocytes), and Flt1 (endothelial cells). (E) Stacked bar plots showing the proportion of each cell type per sample, grouped by age (left), CC line (middle), and sex (right). (F) Box plots of cell type proportions across age groups. Statistical significance was determined using FDR correction with the Benjamini–Hochberg method (FDR < 0.05, **FDR < 0.01, ***FDR < 0.001). (G) Box plots of cell type proportions across CC strains, with significance determined using FDR correction (FDR < 0.05, **FDR < 0.01, ***FDR < 0.001). CC, Collaborative Cross; FDR, false discovery rate OPC, oligodendrocyte precursor cell; snRNA‐seq, single‐nucleus RNA sequencing; UMAP, Uniform Manifold Approximation and Projection.

To characterize the hippocampal transcriptional landscape across genetically diverse aging mice, we profiled hippocampal tissue from eight CC strains across three age points (6, 12, and 18 months) and both sexes. All animals underwent a battery of behavioral assessments for sensorimotor functions including rotarod, inclined screen, and grip strength test, and cognitive performance, including Y‐maze and CFM. CFM was performed prior to tissue collection (Figure 1A). Following quality control and doublet removal, clusters were annotated into eight major cell populations based on the significant enrichment of cluster markers and previously published markers,^33^, ^34^, ^35^ including excitatory neurons, inhibitory neurons, microglia, astrocytes, OPCs, oligodendrocytes, endothelial cells, and a small cluster of unknown identity (Figure 1B). The unknown cluster was excluded from downstream analyses. Cell‐type identities were further supported by a heatmap of differentially expressed marker genes across clusters (Figure S4) and confirmed by the expression of canonical markers, including *Slc17a7* (excitatory neurons), *Gad2* (inhibitory neurons), *Inpp5d* (microglia), *Gja1* (astrocytes), *Pdgfra* (OPCs), *Mog* (oligodendrocytes), and *Flt1* (endothelial cells) projected onto UMAP space (Figure 1C). An extended panel of marker genes, including *Rbfox3*, *Gad1*, *Slc17a6*, *C1qb*, *P2ry12*, *Csf1r*, *Aqp4*, *S1pr1*, *Mbp*, *Plp1*, *Tnr*, *Cspg4*, *Dcn*, and *Pecam1*, further confirmed the specificity of each cell‐type annotation (Figure S5).

Cell‐type proportions were broadly stable across age groups, CC lines, and sexes, with excitatory neurons consistently representing the dominant population (Figure 1D). To assess for compositional differences, we applied an ANOVA model to test the effect of sex, genetic background, and age on each cell type, with FDR correction across all tests. No significant effect of sex was detected for any cell type. In contrast, oligodendrocyte proportions varied significantly by both CC line (*F*[7, 74] = 6.05, FDR < 0.001) and age (*F*[2, 74] = 9.5, FDR = 0.00149), and microglia proportions differed significantly by line (*F*[7, 74) = 7.81, FDR < 0.001) and age (*F*[2, 74) = 6.79, FDR = 0.0103) (Figure 1E,F and Table S2). These results suggest that the overall cellular composition of the hippocampus is largely preserved across conditions, genetic background, and age are the primary determinants of glial cell composition.

### 3.2 Early aging induces cell‐type‐specific transcriptional responses in the hippocampus, with excitatory neurons showing the greatest dysregulation

**FIGURE 2:**
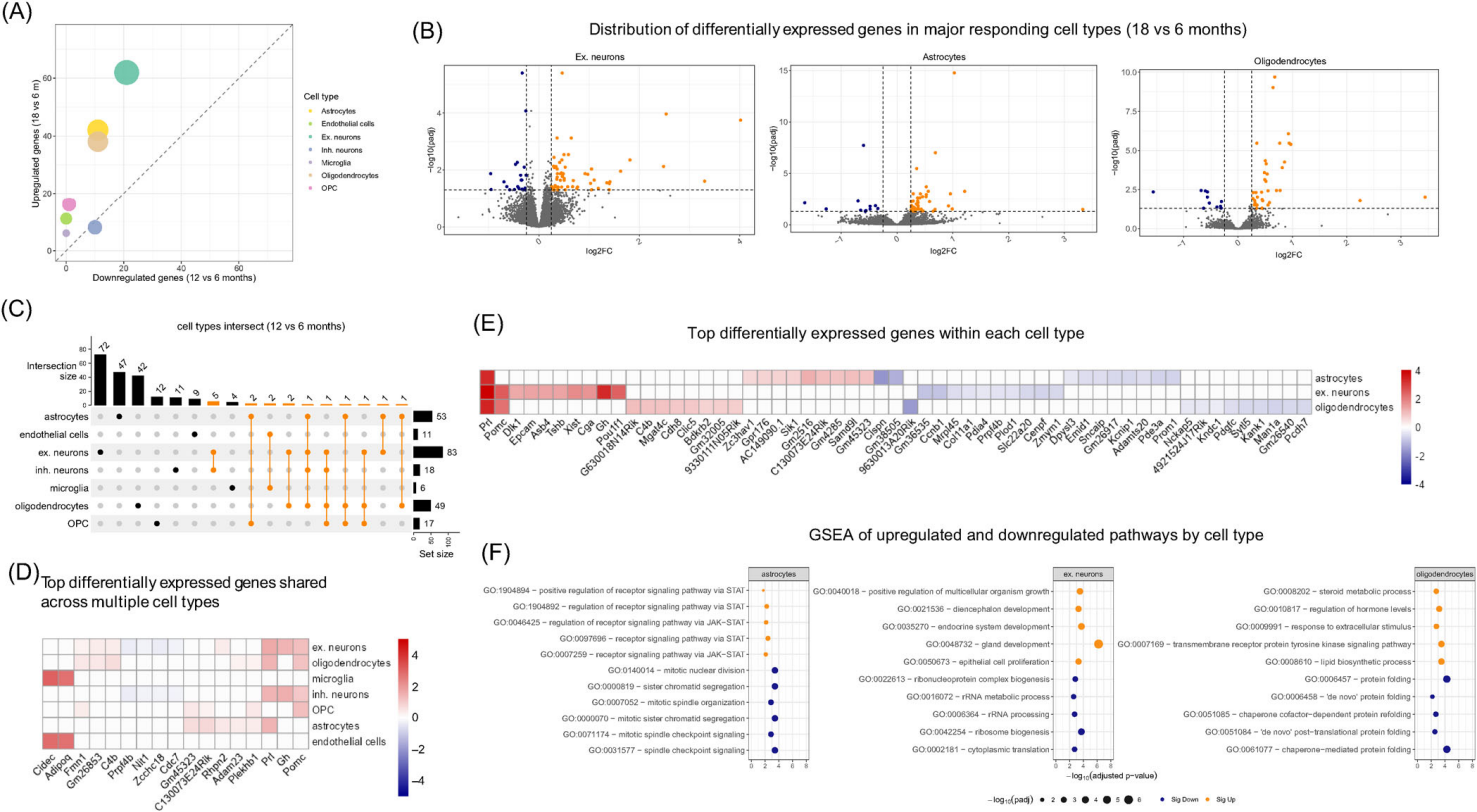
Cell‐type‐specific transcriptional changes during the early stages of chronological aging (12 vs 6 months). (A) Bubble plot summarizing number of DEGs across major hippocampal cell types during early aging (12 vs 6 months); bubble size reflects total DEG counts and color indicates cell type. (B) Volcano plots showing DEG distributions in excitatory neurons, astrocytes, and oligodendrocytes during early aging. Orange indicates genes upregulated in 12 months; blue indicates downregulated genes. Significance was defined as |log_2_FC > 0.25 and FDR < 0.05. (C) UpSet plot of overlap of DEGs across cell types at 12 vs 6 months. (D) Heatmap of DEGs shared across multiple cell types for comparison of 12 versus 6 months. Values are scaled as z‐scores derived from log_2_FC. (E) Heatmap of top DEGs (ranked by absolute log_2_FC) in major responding cell types during early aging. Values are shown as z‐scores of log_2_FC. (F) GSEA showing pathway‐level changes during early aging. Orange points represent top five positively enriched pathways (NES > 0, FDR < 0.05), and blue points represent the top five negatively enriched pathways (NES < 0, FDR < 0.05). DEG, differentially expressed gene; FDR, false discovery rate; GSEA, gene set enrichment analysis; log_2_FC, log_2_ fold change; NES, normalized enrichment score.

To characterize transcriptional changes associated with early aging, we performed pseudobulk differential expression analysis comparing 12‐ to 6‐month‐old mice across all major hippocampal cell types. Excitatory neurons contributed the largest number of DEGs. Following removal of genes with undefined FDR, 83 genes as statistically significant (FDR < 0.05, |log_2_FC| > 0.25), of which 62 were upregulated and 21 were downregulated, followed by astrocytes (upregulated = 42, downregulated: 11) and oligodendrocytes (upregulated: 38, downregulated: 11), while other cell types showed comparatively modest transcriptional responses (Figure 2A,B and Table S3). Intersection analysis revealed that the majority of DEGs were cell‐type specific, with limited overlap across populations (Figure 2C). Examination of DEGs shared across at least two cell types revealed notable outliers including *Cidec* and *Adipoq*, which exhibited exceptionally large effect sizes in both microglia (*Cidec*: log2FC = 20.08, FDR = 5.43e‐05; *Adipoq*: log2FC = 14.77, FDR = 0.044) and endothelial cells (*Cidec*: log2FC = 16.09, FDR = 0.006; *Adipoq*: log2FC = 15.06, FDR = 0.016) (Figure 2D). Other genes consistently dysregulated across multiple cell types included *Prl*, *Gh*, and *Egfem1*. The top DEGs by absolute log2FC within astrocytes, excitatory neurons, and oligodendrocytes are visualized in Figure 2E. Given the relatively modest number of DEGs at this early aging stage, we applied GSEA on the full ranked gene list to capture broader directional transcriptional trends. This revealed enrichment of JAK‐STAT and receptor signaling pathways in astrocytes (GO:0046425, NES = 2.084, FDR = 7.04e‐03), positive regulation of multicellular organism growth and endocrine system development in excitatory neurons (GO:0040018, NES = 2.450, FDR = 3.07e‐04), and protein folding and chaperone‐mediated processes in oligodendrocytes (GO:0061077, NES = −2.435, FDR = 5.27e‐05) (Figure 2F).

### 3.3 Late‐stage aging shifts transcriptional burden to glia with coordinated astrocyte and oligodendrocyte dysregulation

**FIGURE 3:**
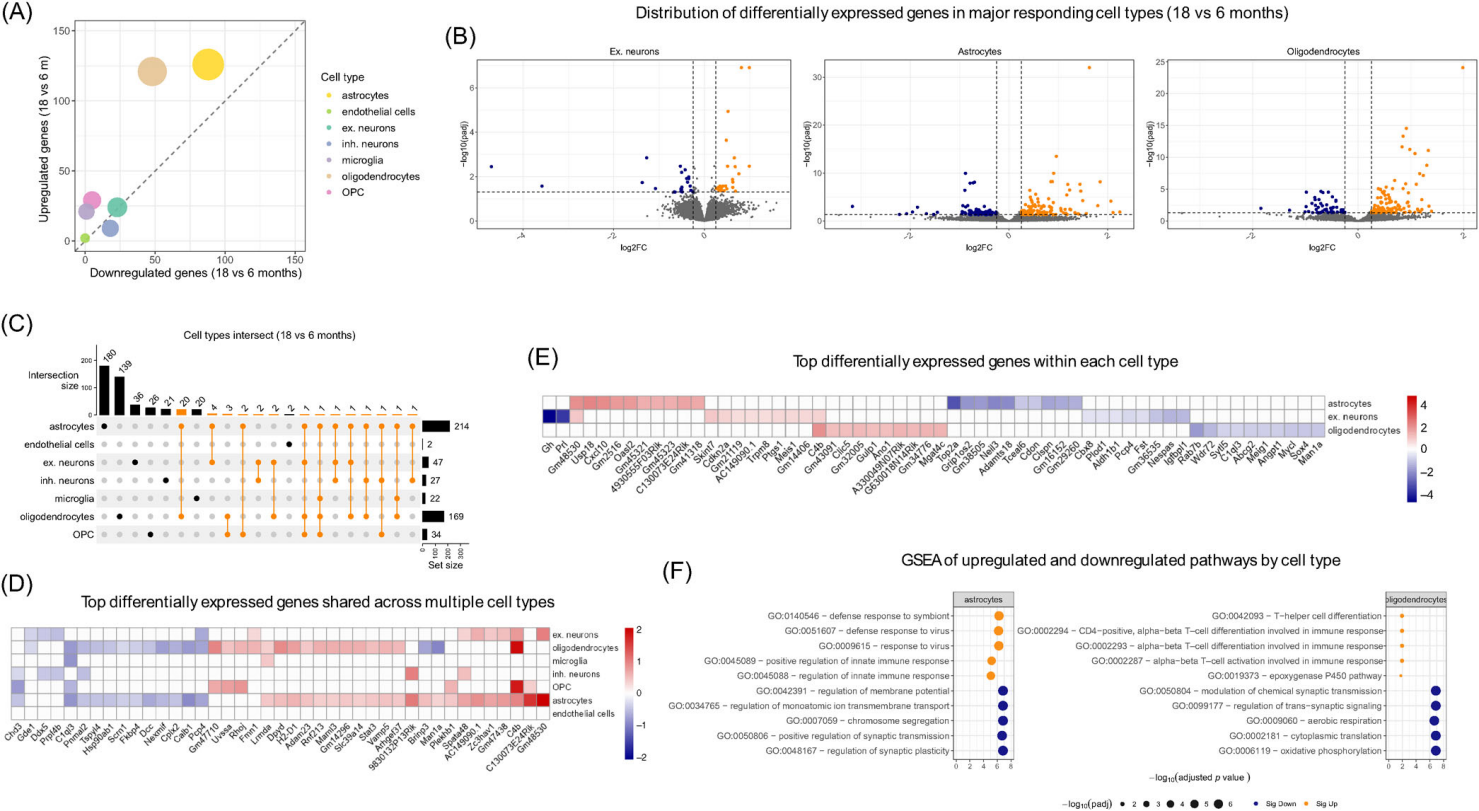
Cell‐type‐specific transcriptional changes during late stages of chronological aging (18 vs 6 months). (A) Bubble plot summarizing DEG counts across major cell types during late aging (18 vs 6 months); bubble size reflects total DEG counts and color indicates cell type. (B) Volcano plots showing DEG distributions in excitatory neurons, astrocytes, and oligodendrocytes during late aging. Orange indicates genes upregulated in 18 months, and blue indicates downregulated genes. Significance was defined as |log_2_FC > 0.25 and FDR < 0.05. (C) UpSet plot of overlap of DEGs across cell types at 18 versus 6 months. (D) Heatmap of DEGs shared across multiple cell types for the 18‐ versus 6‐month comparison. Values are shown as z‐scores of log_2_FC. (E) Heatmap of top DEGs in major responding cell types during late aging. Values are shown as z‐scores of log_2_FC. (F) GSEA showing pathway enrichment during late stage of aging. Orange points represent top five positively enriched pathways (NES > 0, FDR < 0.05), and blue points represent top five negatively enriched pathways (NES < 0, FDR < 0.05). DEG, differentially expressed gene; log_2_FC, log_2_ fold change; FDR, false discovery rate; GSEA, gene set enrichment analysis; NES, normalized enrichment score.

We next characterized transcriptional changes associated with late‐stage aging by comparing 18‐ to 6‐month‐old mice. Strikingly, the landscape of differential expression shifted substantially from early aging: astrocytes (upregulated: 126, downregulated: 88) and oligodendrocytes (upregulated: 121, downregulated: 48) now contributed the largest numbers of DEGs (FDR < 0.05, |log_2_FC. > 0.25), while excitatory neurons (upregulated: 24, downregulated: 23) showed considerably fewer transcriptional changes despite comprising the dominant cell population (Figure 3A,B and Table S4). This shift suggests that glial cells become the primary transcriptional responders during late‐stage hippocampal aging. Intersection analysis revealed largely cell‐type‐specific DEG signatures, but with a notable overlap of 20 genes between astrocytes and oligodendrocytes, where a substantial number of genes were dysregulated concordantly in both cell types (Figure 3C). Among the shared upregulated genes at 18 months, 11 genes were consistently elevated in both populations, while nine were among the concordantly downregulated genes, indicating a coordinated transcriptional response in glial cells during late‐stage aging that may reflect shared adaptive mechanisms between astrocytes and oligodendrocytes (Figure 3D). The top DEGs by absolute log2FC within astrocytes, excitatory neurons, and oligodendrocytes are visualized in Figure 3E. GSEA revealed distinct pathway enrichment profiles between the two major responding cell types. Astrocytes showed upregulation of antiviral and innate immune response pathways (GO:0051607, NES = 1.977, FDR = 6.61e‐07), with concurrent downregulation of synaptic plasticity regulation (GO:0048167, NES = −2.322, FDR = 1.49e‐07). Oligodendrocytes were enriched for T‐helper cell differentiation (GO:0042093, NES = 1.935, FDR = 1.14e‐02), while exhibiting downregulation of oxidative phosphorylation and aerobic respiration (GO:0006119, NES = −2.439, FDR = 1.35e‐07) during late aging (Figure 3F).

**FIGURE 4:**
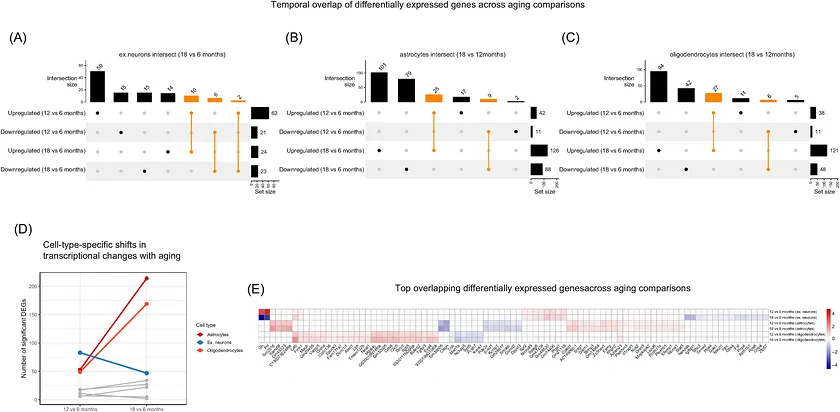
Chronological aging‐associated transcriptional changes show cell‐type‐specific shifts across hippocampus. (A–C) UpSet plots showing directional consistency of DEGs across time points (12 vs 6 months and 18 vs 6 months) for excitatory neurons (A), astrocytes (B), and oligodendrocytes (C). (D) Line plot summarizing temporal transcriptional trajectories across cell types, showing early responses in excitatory neurons and progressive changes in astrocytes and oligodendrocytes. (E) Heatmap of overlapping DEGs across aging comparisons. Values are shown as z‐scores of log_2_FC. DEG, differentially expressed gene; log_2_FC, log_2_ fold change.

To further examine the dynamics of transcriptional change across chronological aging, we examined the directional consistency of DEGs across time points within excitatory neurons, astrocytes, and oligodendrocytes using UpSet plots (Figure 4A–C). Each cell type includes subsets of genes consistently up‐ or downregulated across both comparisons. Notably, excitatory neurons show two genes with opposing trajectories between time points. Oligodendrocytes and astrocytes accumulated transcriptional changes progressively from early to late aging, whereas excitatory neurons showed an early transcriptional response that diminished substantially at later time points (Figure 4D).

Comparison of overlapping DEGs between the 12‐ versus 6‐month and 18‐ versus 6‐month contrasts revealed distinct temporal expression patterns across cell types (Figure 4E). Most notably, *Gh* and *Prl* were strongly upregulated in excitatory neurons at 12 months but showed marked downregulation at 18 months. Consistent with this pattern, direct comparison of 18‐ versus 12‐month mice revealed only minimal transcriptional changes overall; however, *Prl* and *Gh* were consistently downregulated across nearly all cell types examined, including OPCs, astrocytes, excitatory neurons, inhibitory neurons, and microglia (Figure S6). In addition, *Adam23* was consistently upregulated across both time points in astrocytes and oligodendrocytes, while *C4b* and *Fmn1* were consistently upregulated across both time points in excitatory neurons and oligodendrocytes. Given the frequent emphasis on microglia in neurodegenerative disease, we additionally examined the hippocampal myeloid compartment at higher resolution using MapMyCells reference mapping.^36^ This analysis resolved parenchymal microglia and border‐associated macrophages (BAMs) but did not identify additional inflammatory or disease‐associated microglial populations at the resolution supported by our dataset. Because BAMs were relatively sparse, the two myeloid populations were combined in the primary analysis; however, we also evaluated them independently to determine whether population heterogeneity masked age‐associated transcriptional changes. BAMs showed no significant DEGs in any chronological‐aging comparison. Parenchymal microglia likewise exhibited limited differential expression, with one upregulated gene at 12 versus 6 months, two downregulated genes at 18 versus 12 months, and 22 DEGs at 18 versus 6 months, including 21 upregulated genes and one downregulated gene (Table S5). Thus, separating the myeloid compartment did not reveal a broad inflammatory transcriptional response to chronological aging in the hippocampus.

Taken together, these findings demonstrate that chronological aging in the hippocampus is characterized by divergent cell‐type‐specific transcriptional trajectories, with excitatory neurons exhibiting early but transient responses and glial populations, particularly astrocytes and oligodendrocytes, driving sustained transcriptional remodeling across aging. Notably, microglial transcriptional changes were minimal across both comparisons, suggesting that neuroinflammatory microglial activation is not a prominent feature of physiological aging in the hippocampus.

### 3.4 Cognitive decline–associated transcriptional changes are driven by excitatory neurons and with little overlap with chronological aging

We next characterized gene signatures associated with cognitive decline/resilience. To characterize cognitive aging across genetically diverse backgrounds, CFM was assessed in a cohort of 208 mice spanning eight CC strains (CC003, CC004, CC005, CC006, CC019, CC032, CC041, and CC061), across three age points (6, 12, and 18 months) and both sexes. It should be noted that the cohort was not fully balanced across all conditions: strains CC006 and CC041 lacked 18‐month time points, and CC019 had limited 18‐month representation (*n* = 3). A subset of these animals (*n* = 107) was selected for snRNA‐seq, with sequencing samples balanced as evenly as feasible across sex and age within each strain (Table S6).

**FIGURE 5:**
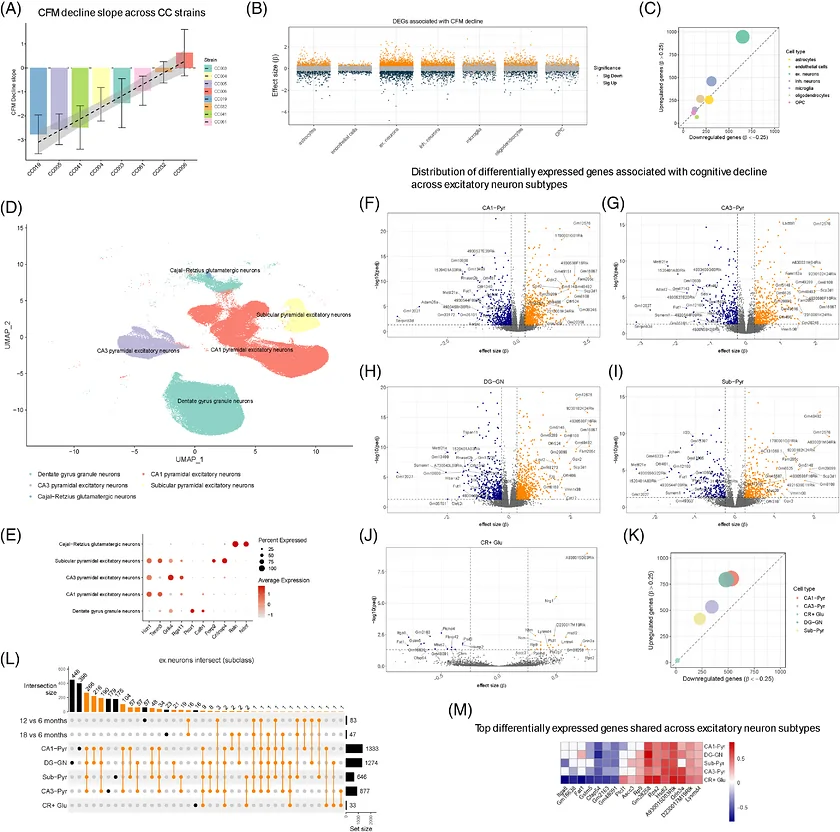
Cognitive decline–associated transcriptional changes are driven by excitatory neurons and with limited overlap with chronological aging. (A) CFM decline slope across CC strains. Bars represent estimated decline slopes for each strain, with error bars indicating mean ± SEM. The dashed line indicates the overall linear trend across strains, with the shaded area representing the 95% confidence interval. (B) Scatter plot showing distributions of DEGs across major cell types. Orange indicates genes positively associated with cognitive performance (i.e., less decline), and blue indicates genes negatively associated with cognitive performance (greater decline). Significance was defined as |β. > 0.25 and FDR < 0.05. (C) Bubble plot summarizing number of DEGs across cell types; bubble size reflects total DEG counts and color indicates cell type. (D) UMAP visualization of reclustered excitatory neuron subtypes, including CA1 pyramidal neurons (CA1‐Pyr), CA3 pyramidal neurons (CA3‐Pyr), dentate gyrus granule neurons (DG‐GN), subicular pyramidal neurons (Sub‐Pyr), and Cajal–Retzius glutamatergic neurons (CR+ Glu). (E) Dot plot showing expression of canonical marker genes across excitatory neuron subtypes (CA1‐Pyr, CA3‐Pyr, DG‐GN, Sub‐Pyr, and CR+ Glu). Dot size represents the percentage of cells expressing each gene, with color intensity scaled to average expression. (F–J) Volcano plots showing DEGs associated with cognitive decline within each excitatory neuron subtype including CA1‐Pyr (F), CA3‐Pyr (G), DG‐GN (H), Sub‐Pyr (I), and CR+ Glu (J). Orange indicates genes positively associated with decline, and blue indicates negatively associated genes. Significance was defined as |β. > 0.25 and FDR < 0.05. (K) Bubble plot summarizing number of upregulated and downregulated DEGs across excitatory neuron subtypes. Bubble size reflects DEG counts. (L) UpSet plot showing overlap between DEGs associated with cognitive decline and those associated with chronological aging (12 vs 6 months and 18 vs 6 months) across excitatory neuron subtypes. (M) Heatmap of DEGs shared across all excitatory neuron subtypes. Values are shown as z‐scores of log_2_FC. CA1‐Pyr, CA1 pyramidal neuron; CA3‐Pyr, CA3 pyramidal neuron; CR+ Glu, Cajal–Retzius glutamatergic neuron; DG‐GN, dentate gyrus granule neuron; CC, Collaborative Cross; CFM, contextual fear memory; DEG, differentially expressed gene; FDR, false discovery rate; log_2_FC, log_2_ fold change; SEM, standard error of the mean; Sub‐Pyr, subicular pyramidal neuron; UMAP, Uniform Manifold Approximation and Projection.

CFM was assessed terminally prior to tissue harvest. To confirm that the other behavioral measures performed do not confound interpretation of CFM, we examined their correlations with CFM performance. Spearman correlation analysis revealed that Y‐maze spontaneous alternation (rho = 0.0307, *p* = 0.6536) and grip strength (rho = −0.0326, *p* = 0.6105) showed no significant correlation with CFM, while inclined screen (rho = 0.2153, *p* = 0.0007) and rotarod latency to fall (rho = 0.2117, *p* = 0.0008) exhibited only weak positive correlations (Figure S7). To derive a continuous measure of cognitive decline for each strain, we fitted a linear model for each strain separately, extracting the age coefficient as the strain‐specific CFM decline slope. This approach accounts for sex as a covariate, isolating the age‐dependent trajectory of cognitive performance within each genetic background. Strains exhibited substantial variation in their decline trajectories (Figure 5A): CC019, CC005, and CC041 showed the steepest negative slopes, indicating substantial age‐associated cognitive decline, while CC032 and CC006 showed slopes near or above zero. This strain‐specific decline rate was subsequently used as a continuous trait in regression‐based differential expression analysis to identify genes whose expression is associated with cognitive decline trajectory across strains. To identify genes whose expression changes with cognitive decline trajectory, we performed pseudobulk differential expression analysis across all major cell types. Genes with |β. > 0.25 and FDR < 0.05 were considered significant. In this framework, upregulated genes are positively associated with cognitive resilience – higher expression corresponds to better preserved cognitive function – while downregulated genes are those whose reduced expression is associated with faster cognitive decline.

This analysis revealed widespread cognitive decline‐associated transcriptional changes across all cell types examined (Figure 5B and Table S7). Excitatory neurons contributed by far the largest number of significant DEGs in both directions (upregulated: 946, downregulated: 651), followed by inhibitory neurons (upregulated: 459, downregulated: 309) and astrocytes (upregulated: 256, downregulated: 283), while endothelial cells and OPCs showed the fewest cognitive decline‐associated changes (Figure 5C).

Although microglia showed limited transcriptional changes during chronological aging, their established roles in synaptic pruning, activity‐dependent synaptic remodeling, and memory regulation^37^, ^38^, ^39^ motivated us to examine whether microglial gene expression was associated with cognitive decline. To address this, we revisited the myeloid compartment at higher resolution by analyzing the previously identified microglial and BAM populations separately. BAMs showed little association with cognitive decline, with only one significant gene. In contrast, parenchymal microglia showed 371 cognitive decline‐associated DEGs (upregulated: 166, downregulated: 207) (Table S8). Although this response was more extensive than the age‐associated microglial signature, it remained substantially smaller than the cognitive trajectory‐associated signature observed in excitatory neurons. Functional enrichment did not reveal a coherent pro‐inflammatory response. Positive‐associated genes were enriched for processes including cell‐junction organization (GO:0034330, FDR = 2.75 × 10^−6^, z‐score = 6.2), cell adhesion (GO:0007155, FDR = 1.27 × 10^−4^, z‐score = 4.7), and response to peptidoglycan (GO:0032494, FDR = 7.49 × 10^−5^, z‐score = 10.1), whereas negative‐associated genes were enriched for cytokine–cytokine receptor interactions (mmu04060, FDR = 1.17 × 10^−4^, z‐score = 5.1), regulation of leukocyte migration (GO:0002685, FDR = 1.54 × 10^−4^, z‐score = 5.1), and transforming growth factor‐beta signaling (R‐MMU‐2173791, FDR = 1.63 × 10^−4^, z‐score = 8.7) (Figure S8). Thus, although immune‐related pathways were represented in both directions, the microglial signature was more consistent with modulation of homeostatic, signaling, and immune‐regulatory functions than with widespread pro‐inflammatory activation.

Given the prominent contribution of excitatory neurons to cognitive decline–associated transcriptional changes and their established role in hippocampal circuits underlying CFM, we focused subsequent analyses on this population. Excitatory neurons were subset and re‐clustered at a resolution of RNA_snn_res = 0.01, yielding five transcriptionally distinct subclusters. Cell identities were assigned using MapMyCells with reference to the Allen Brain Atlas,^36^ resulting in annotation of CA1 pyramidal (CA1‐Pyr), CA3 pyramidal (CA3‐Pyr), dentate gyrus granule (DG‐GN), subicular pyramidal (Sub‐Pyr), and Cajal–Retzius glutamatergic (CR+ Glu) neurons (Figure 5D). Subtype assignments were further supported by the expression of canonical region‐specific marker genes across clusters (Figure 5E).

Pseudobulk differential expression analysis was performed within each excitatory neuron subtype using the cognitive decline slope as a continuous variable. This analysis identified substantial CFM decline–associated transcriptional changes across CA1‐Pyr, DG‐GN, CA3‐Pyr, and Sub‐Pyr neurons, whereas CR+ glutamatergic neurons showed few significant DEGs (Figure 5F–J). Among these subtypes, CA1‐Pyr and DG‐GN exhibited the greatest number of decline‐associated DEGs (CA1‐Pyr: 804 upregulated, 529 downregulated; DG‐GN: 793 upregulated, 481 downregulated), followed by CA3‐Pyr (533 upregulated, 344 downregulated) and Sub‐Pyr (418 upregulated, 228 downregulated) (Figure 5K and Table S9).

While many DEGs were subtype‐specific, there was also notable overlap across subtypes. A total of 266 DEGs were shared among all major excitatory neuron populations except for CR+ glutamatergic neurons, with substantial overlap between CA1‐Pyr and DG‐GN, including 216 genes common to both subtypes. In addition, a small core set of DEGs was consistently detected across all excitatory neuron subtypes (four downregulated and five upregulated genes) (Figure 5M). Together, these results indicate that CA1‐Pyr and DG‐GN neurons represent the primary transcriptional responders to cognitive decline within the hippocampal excitatory neuron population.

Because chronological aging and cognitive decline occur over similar timeframes, we next examined whether decline‐associated transcriptional changes simply reflected general aging‐related gene expression shifts or represented a distinct molecular program. To address this, we compared cognitive decline–associated DEGs in excitatory neurons with those identified in chronological aging contrasts (12 vs 6 months and 18 vs 6 months). Across all subtypes, the majority of cognitive decline–associated DEGs were unique to cognitive decline and showed minimal overlap with aging‐associated DEGs from either comparison (Figure 5L and Figure S9). These findings suggest that cognitive decline and chronological aging are largely driven by distinct transcriptional programs in excitatory neurons and that the gene expression changes associated with CFM decline cannot be explained by general age‐related shifts.

**FIGURE 6:**
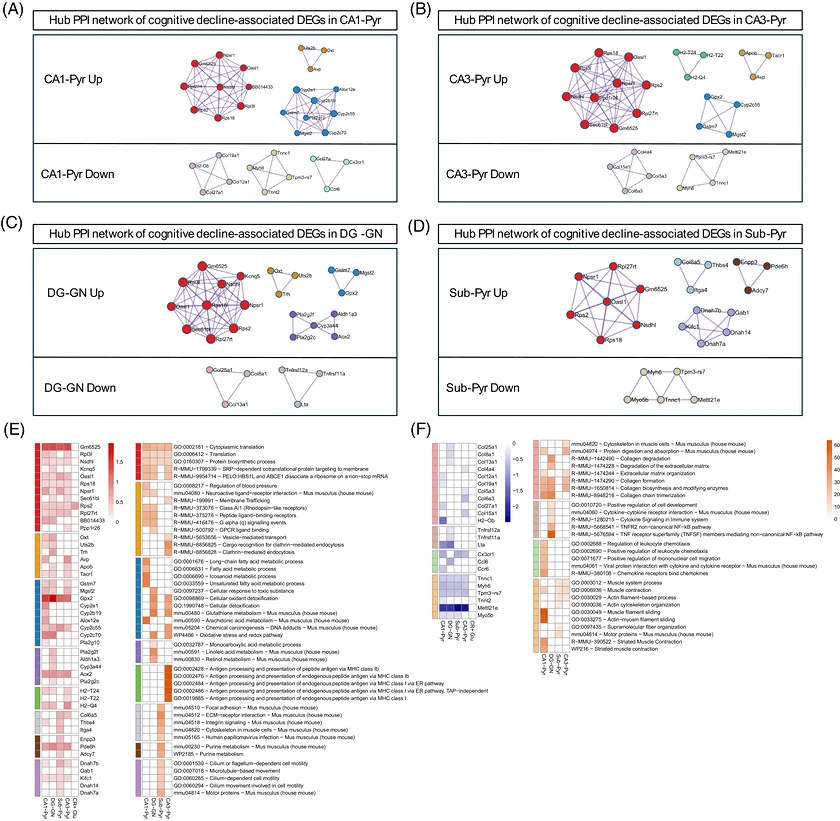
Excitatory neuron PPI modules associated with cognitive decline. (A–D) Hub PPI networks constructed from significant cognitive decline‐associated DEGs within excitatory neuron subtypes, including CA1‐Pyr (A), CA3‐Pyr (B), DG‐GN (C), and Sub‐Pyr (D). Separate networks are shown for upregulated and downregulated DEGs within each subtype. Nodes represent genes, and edges represent known or predicted protein–protein interactions based on STRING. Edge thickness reflects interaction confidence. Node colors indicate PPI network modules enriched for shared biological processes. (E) Heatmap of significantly upregulated DEGs and corresponding enriched pathways across excitatory neuron subtypes associated with cognitive decline. Genes are grouped according to PPI network modules (colors correspond to module assignments shown in panel H). For the gene expression heatmap (left), values are shown as z‐scores of log_2_FC. For the pathway enrichment heatmap (right), color intensity reflects enrichment scores (Metascape z‐scores). (F) Heatmap of significantly downregulated DEGs and corresponding enriched pathways across excitatory neuron subtypes associated with cognitive decline. Genes are grouped according to PPI network modules (colors correspond to module assignments shown in panel H). For the gene expression heatmap (left), values are shown as z‐scores of log_2_FC. For the pathway enrichment heatmap (right), color intensity reflects enrichment scores (Metascape z‐scores). CA1‐Pyr, CA1 pyramidal neuron; CA3‐Pyr, CA3 pyramidal neuron; DG‐GN, dentate gyrus granule neuron; DEG, differentially expressed gene; PPI, protein–protein interaction; STRING, Search Tool for the Retrieval of Interacting Genes/Proteins; Sub‐Pyr, subicular pyramidal neuron; log_2_FC, log_2_ fold change.

Given the large number of CFM decline–associated DEGs identified in each excitatory neuron subtype, we next prioritized biologically relevant candidates by examining PPI networks. For each subtype, upregulated and downregulated genes were analyzed separately, and the resulting primary PPI networks are shown in Figures S10–S16. We focused on genes that occupy highly connected positions within these networks and that recur within coherent modules across multiple subtypes, as these are more likely to represent core mechanisms underlying cognitive decline rather than isolated transcriptomic changes. From these networks we identified several recurrent modules shared across CA1‐Pyr, CA3‐Pyr, DG‐GN, and Sub‐Pyr neurons (Figure 6A–D). Among upregulated genes, a prominent module centered on ribosomal and translational components (e.g., *Rps2*, *Rps18*, *Gm6525*, *Nsdhl*, *Oasl1*, *Npsr1*, *Rpl27rt*), along with genes involved in glutathione metabolism (*Gstm7*, *Mgst2*, *Gpx2*), was consistently observed across all four subtypes. In contrast, downregulated modules were enriched for structural and extracellular matrix‐related genes, including collagen‐associated genes, cytoskeletal components (e.g., *Mettl21e*, *Myh6*, *Tnnc1*, *Tpm3‐rs7*), and immune‐related genes (*Cx3cr1* and *Lta*). Together, these cross‐subtype network patterns highlight a set of high‐confidence candidate genes and pathways for further functional investigation. To further define the biological functions of the co‐expression modules, we compiled genes from the PPI networks and visualized their log2 fold change across subtypes alongside Metascape enrichment results. For upregulated modules, the most consistently elevated module across one or more subtypes was strongly enriched for cytoplasmic translation (GO:0002181, FDR = 2 × 10^−6^, z‐score = 20) and protein biosynthetic processes (GO:0160307, FDR = 1.26 × 10^−4^, z‐score = 12). Additional modules highlighted pathways related to neuroactive ligand–receptor interaction (mmu04080, FDR = 1.58 × 10^−3^, z‐score = 13), glutathione metabolism (mmu00480, FDR = 1 × 10^−4^, z‐score = 30), and oxidative stress and redox pathway (WP4466, FDR = 1.58 × 10^−4^, z‐score = 27) (Figure 6E). In contrast, downregulated modules showed a distinct set of functional enrichments. The most consistently downregulated modules across one or more subtypes were strongly enriched for extracellular matrix organization (R‐MMU‐1474244, FDR = 1.58 × 10^−3^, z‐score = 14), collagen‐related processes (R‐MMU‐1650814, FDR = 1 × 10^−4^, z‐score = 29), and actin filament‐based processes (GO:0030029, FDR = 2.51 × 10^−4^, z‐score = 12) (Figure 6F).

## 4 DISCUSSION

In this study, we used hippocampal snRNA‐seq across genetically diverse CC mice to identify transcriptional signatures associated with chronological aging from those associated with cognitive decline. A central finding of this study is that chronological aging and cognitive decline are molecularly dissociable processes within the hippocampus. Although aging remains the strongest population‐level risk factor for cognitive impairment,^40^ the transcriptional signatures associated with chronological aging were relatively modest and largely cell‐type specific, whereas those associated with cognitive decline were broader, stronger, and predominantly localized to excitatory neurons. This distinction suggests that cognitive decline is not simply as an extension of normal age‐related transcriptional drift. Rather, it appears to involve additional molecular reprogramming that is selectively engaged in specific neuronal populations.

This interpretation is consistent with a prior single‐cell study showing that brain aging does not follow a single uniform transcriptional trajectory but instead drives distinct responses across neuronal and glial cell types.^41^ We hypothesize that chronological aging progressively reduces cellular homeostatic capacity, whereas cognitive decline emerges when accumulated molecular stress exceeds the compensatory capacity of neural circuits. Age‐associated changes in mitochondrial energetics, redox regulation, proteostasis, synaptic maintenance, and glial support may gradually reduce neuronal reserve, while genetic background influences both the rate at which these changes accumulate and the ability of hippocampal neurons to compensate. Individuals of the same chronological age may therefore differ in biological brain age and cognitive trajectory. This hypothesis is consistent with studies showing that aging proceeds asynchronously across organs and cell types.^42^, ^43^ Organ‐specific plasma proteomic clocks further associate accelerated brain and vascular aging with cognitive decline and AD progression,^44^ while cell‐type‐specific transcriptomic clocks indicate partially non‐overlapping aging signatures among human brain cell populations.^45^ Although these studies do not establish that the brain ages independently, they support variation in biological brain aging as one contributor to heterogeneous cognitive outcomes.

Our chronological‐aging analysis supports a staged response across hippocampal cell populations: Excitatory neurons showed the largest early transcriptional signature, whereas astrocytes and oligodendrocytes accounted for more changes at later ages. A similar shift toward stronger glial involvement has been described in recent single‐cell studies of aging and neurodegeneration, in which astrocytic and oligodendroglial states become increasingly prominent in later or more stressed conditions.^11^ In contrast, microglial transcriptional changes were limited in both aging comparisons, and the overall pattern did not support a broad disease‐associated inflammatory program. Given the frequent emphasis on microglia in neurodegenerative disease, these findings suggest that widespread microglial activation is not a dominant feature of physiological aging in the hippocampus. However, this does not exclude the possibility of rare, transient, or region‐specific microglial states that were not detected at the resolution of our dataset. Studies of normal mouse aging have reported small interferon‐responsive or DAM1‐like subsets rather than uniform activation, whereas amyloid models show stronger plaque‐associated disease‐associated microglial programs.^19^, ^34^, ^42^, ^46^ Thus, the microglial signal in this non‐transgenic hippocampal cohort is more consistent with modulation of homeostatic and immune‐regulatory functions than with widespread pro‐inflammatory activation. Together, these observations emphasize that microglial transcriptional states are highly dependent on brain region and pathological context. The microglial profile in this non‐transgenic hippocampal cohort is therefore more consistent with modulation of homeostatic and immune‐regulatory functions than with widespread pro‐inflammatory activation.

In contrast, variation in cognitive trajectory produced the most extensive transcriptional associations, with CA1 pyramidal and DG‐GN neurons acting as the principal responders. This pattern may partly reflect the close functional relationship between these populations and CFM, which depends on recruitment and reactivation of excitatory ensembles in the dentate gyrus and CA1.^47^, ^48^ Among the pathways emerging from the transcriptional signatures associated with cognitive decline, two were particularly prominent: proteostasis and glutathione/redox metabolism. Within upregulated modules across excitatory neuron subtypes, genes including *Rps2*, *Rps18*, *Gm6525*, *Nsdhl*, *Oasl1*, *Npsr1*, and *Rpl27rt* were enriched for ribosomal proteins, cytoplasmic translation, and protein biosynthesis. Strikingly, these candidates shared the resilience PPI network enriched in aminoacyl‐tRNA biosynthesis that we previously identified in the AD‐BXD population^24^ (Figure S17), suggesting a conserved proteostatic mechanism exists across different genetic backgrounds, and cognitively declining neurons are likely under increased proteostatic demand. One interpretation is that these neurons are attempting to compensate for accumulating protein damage or impaired protein turnover by increasing translational capacity. However, increased protein synthesis in the absence of adequate folding and degradation capacity may also intensify proteostatic stress.^49^ This is consistent with the broader literature showing that neuronal resilience and vulnerability are closely linked to the ability to maintain protein homeostasis. In The Regligious Order Study and Memory and Aging Project (ROSMAP) prefrontal cortex, candidate genes, including HES4, PDE10A, RPH3A, ST6GAL2, and UST, were found to be associated with at least one cognitive resilience measure across multiple excitatory neuronal subtypes.^50^ Similarly, Castanho et al. identified MEF2C, ATP8B1, and RELN as markers of resilient neuronal populations. Resilient excitatory neurons also showed selective upregulation of Hsp40, Hsp70, and Hsp110 chaperone‐family members.^51^ Although these genes were not among the primary candidates identified in our CC dataset, the two studies converge at the pathway level through the enrichment of translation, protein folding, and proteostasis. The differences in individual genes likely reflect differences in brain region, cell state, pathological burden, genetic background, and the operational definition of cognitive resilience. Additionally, a foundational study characterizing the aging mouse brain at single‐cell resolution did not directly assess cognitive function but provided important pathway‐level context for our findings. It identified ribosomal and translation‐associated programs that varied across cell types, including genes represented in our cognitive decline–associated modules such as Rps2 and Rps18.^41^ Collectively, these comparisons suggest partial cross‐species and cross‐study convergence in the implication of excitatory neurons and proteostasis‐related processes, while emphasizing that the direction and gene‐level composition of these programs remain context dependent.

Glutathione and redox regulation also connect the present findings with our previous AD‐BXD work. Hippocampal Gsr was previously nominated through genetic mapping and proteomics as a candidate resilience factor; higher GSR protein abundance was associated with resilience to the cognitive effects of 5XFAD, and a cis‐pQTL for Gsr was also linked in trans to the abundance of multiple mitochondrial and synaptic proteins.^52^ Higher brain GSR transcript abundance was associated with more favorable cognitive and AD‐pathology outcomes in ROSMAP.^53^ Although Gsr itself was not a major cognitive decline–associated gene in the CC population, Gpx2, Gstm7, Mgst2, and related redox pathways were implicated across excitatory neuronal subtypes. We interpret this as pathway‐level convergence rather than direct replication of Gsr‐associated molecular changes. This pathway is also of particular translational interest, as therapeutic strategies targeting glutathione metabolism have been explored to improve cognitive function. GlyNAC, a combination of glycine and N‐acetylcysteine, has been investigated as a means of increasing glutathione availability and enhancing mitochondrial function. Both clinical and preclinical studies have reported improvements in glutathione deficiency, oxidative stress, and mitochondrial dysfunction, with some evidence for benefits in cognitive or brain‐related outcomes.^54^, ^55^, ^56^, ^57^, ^58^ Taking together, this convergence of evidence across different animal models is important. The CC cohort captures variability in cognitive aging in the absence of AD mutations, while our previous AD‐BXD population study investigated cognitive resilience in the context of familial AD pathology. Despite these biological differences, both systems implicate neuronal metabolic stability, oxidative stress defense, and proteostasis as key components of preserved cognitive function. This overlap strengthens the interpretation that these pathways reflect a conserved molecular basis of cognitive resilience across diverse genetic backgrounds.

Several limitations should be considered in this study. First, although sex differences in the trajectory of cognitive decline and aging have been reported,^59^, ^60^ our current dataset was not sufficiently powered to systematically assess sex‐specific effects within each CC strain. As a result, potential sex‐dependent transcriptional responses may be underrepresented. Second, while the CC population captures substantial genetic diversity, our sample size limits the statistical power to identify specific genetic loci associated with cognitive decline or to perform robust expression quantitative trait locus (eQTL) mapping.^61^, ^62^ Consequently, the genetic drivers underlying the observed transcriptional signatures remain to be defined. Future studies with larger cohorts and expanded sampling will be necessary to resolve these genetic contributions and to better capture sex‐specific effects on cognitive aging.

In summary, our data indicate that chronological aging and cognitive trajectory are associated with largely separable hippocampal transcriptional patterns. Chronological aging was characterized by relatively modest and staged cell‐type‐specific remodeling, with earlier responses in excitatory neurons and more sustained transcriptional changes in astrocytes and oligodendrocytes at later ages. In contrast, cognitive decline was defined by a much stronger excitatory neuron‐centered transcriptional signatures enriched for proteostasis, translation, oxidative stress, and glutathione‐related pathways. The convergence of these findings with previous AD‐BXD work, particularly the association of resilience with GSR and redox homeostasis, supports a model in which maintenance of protein homeostasis and antioxidant capacity is central to preserved cognitive function.

## CONFLICT OF INTEREST STATEMENT

Catherine C. Kaczorowski serves on advisory boards, scientific committees, and governance boards related to aging and Alzheimer's disease research, including the National Institute on Aging (NIA), the Jackson Laboratory (JAX), the Alzheimer's Disease Sequencing Project (ADSP), the Cognitive Aging Summit IV Planning Committee, and the American Federation for Aging Research (AFAR) board of directors. Catherine C. Kaczorowski is advisory member of NIA Reserve and Resilience Non‐Human Studies Workgroup, scientific advisor of Resilience/Resistance Against Alzheimer's Disease in Centenarians and Offspring, and scientific advisor of Functional Genomics Consortium at NIA, external advisory board, external advisory board member of Special Mouse Strains Resource P40OD011102 at JAX, advisor of ADSP, member of Cognitive Aging Summit IV, Planning Committee at National Institutes of Health (NIH), and member of the AFAR board of directors. Catherine C. Kaczorowski is a co‐inventor on the patent “DLGAP2 as a Therapeutic Target for Alzheimer's Disease and Age‐Related Cognitive Decline” (WO2020092862A1; PCT/US2019/059311; current assignee: Jackson Laboratory). The remaining authors declare no competing interests. Author disclosures are available in the Supporting Information.

## CONSENT STATEMENT

This study involved only animal subjects. All procedures were approved by the Institutional Animal Care and Use Committees and conducted in accordance with relevant guidelines and regulations.

## Supporting information




**Supporting file 1**: alz71825‐sup‐0001‐Figures.pdf

**Supporting file 2**: alz71825‐sup‐0002‐Tables.xlsx
