# Supplementary material for: Hippocampal snRNA‐seq in Collaborative Cross reveals molecular signatures of cognitive resilience independent of chronological aging
Source: Alzheimers Dement. 2026 Sep 16;22(9):e71825. doi: 10.1002/alz.71825 (PMC13583060; doi:10.1002/alz.71825)

Supplementary Figure 1

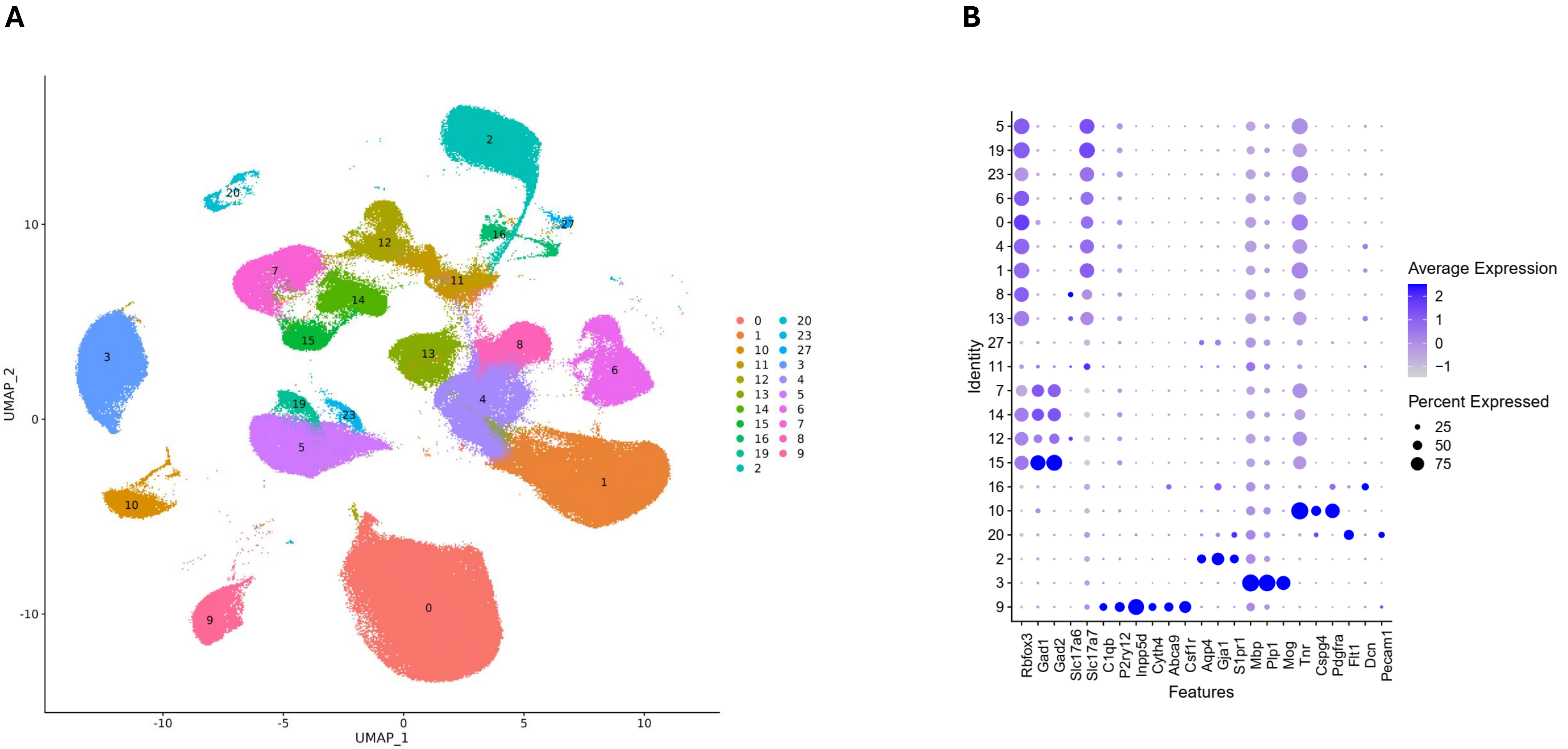

Supplementary Figure 2

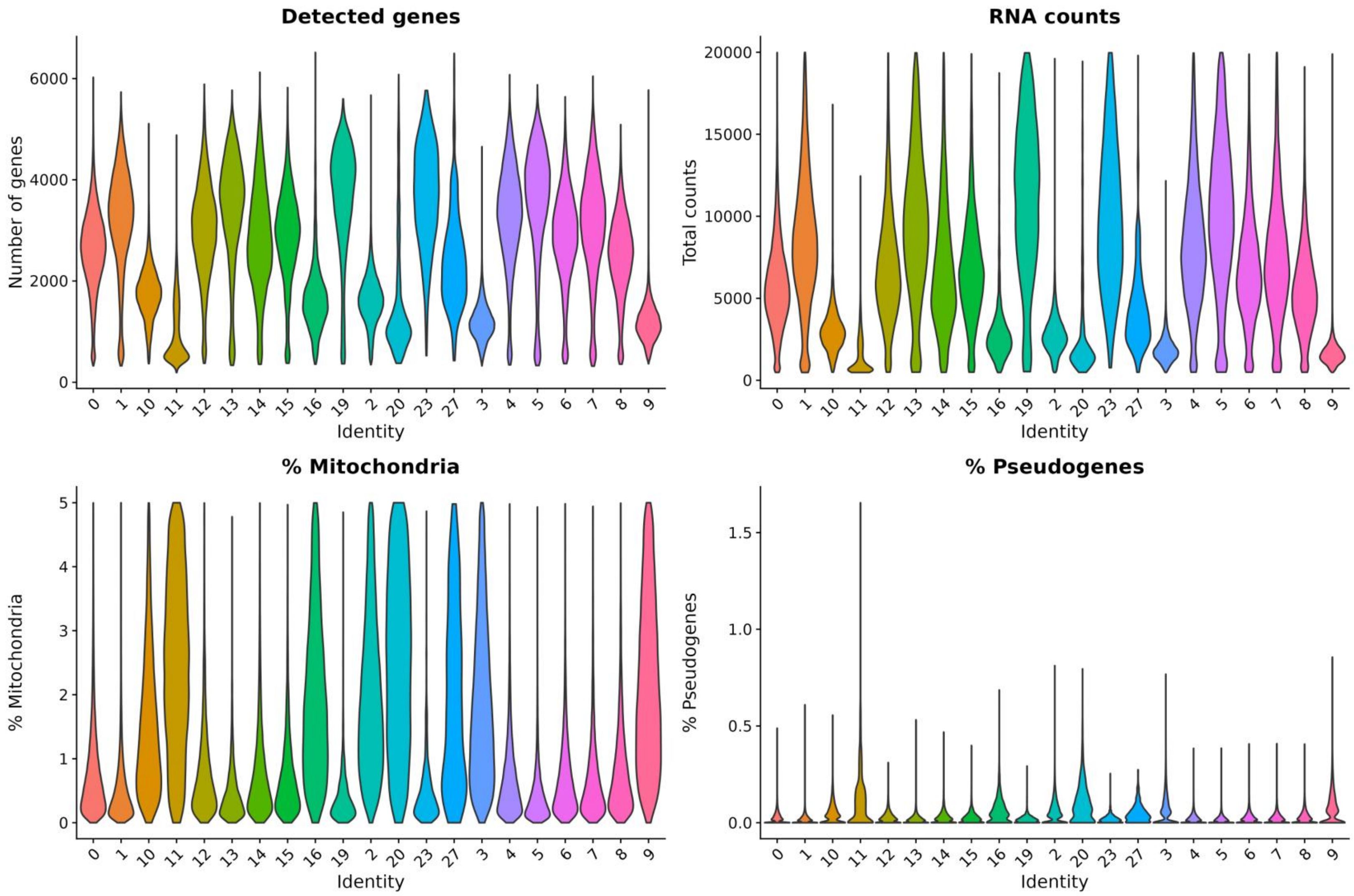

Supplementary Figure 3

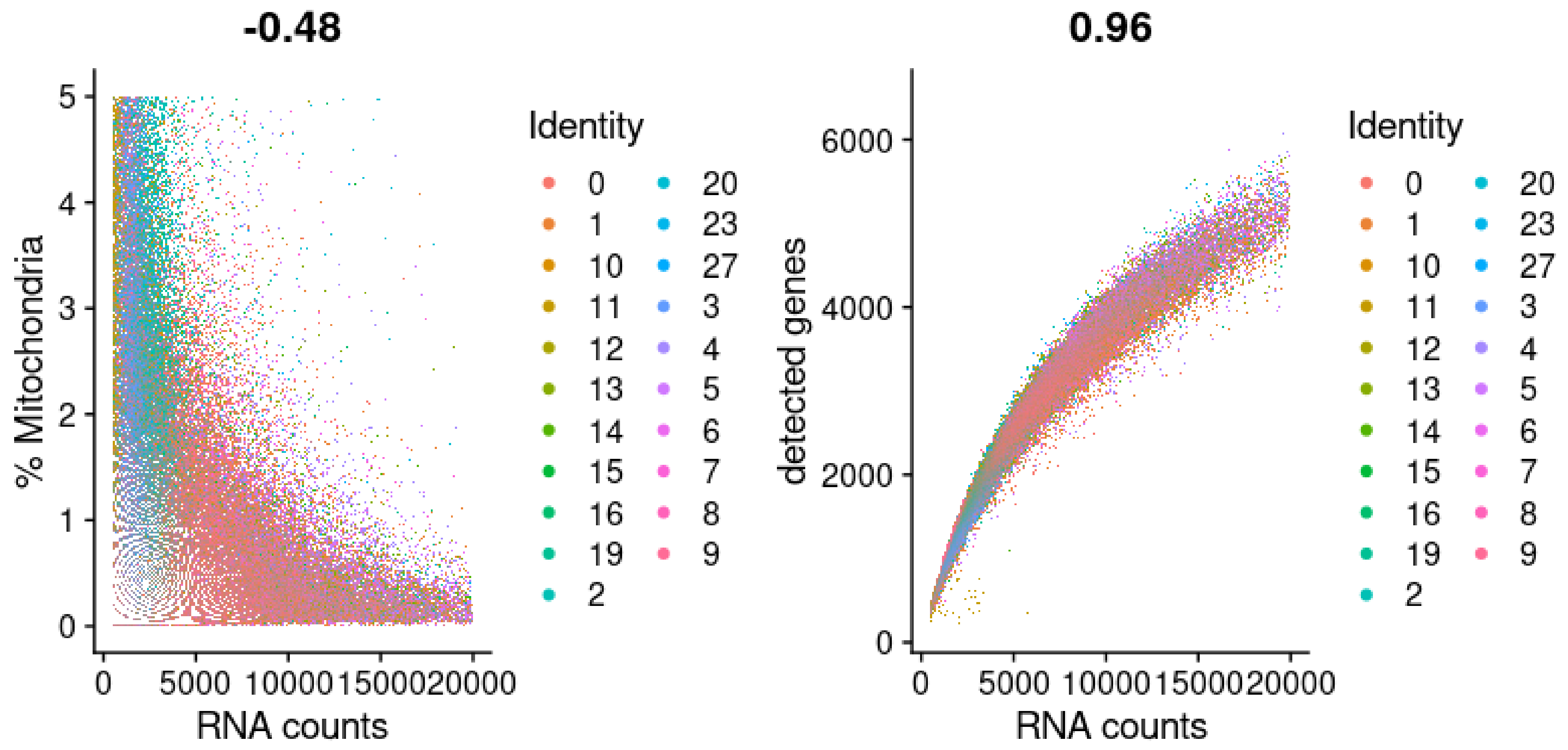

Supplementary Figure 4

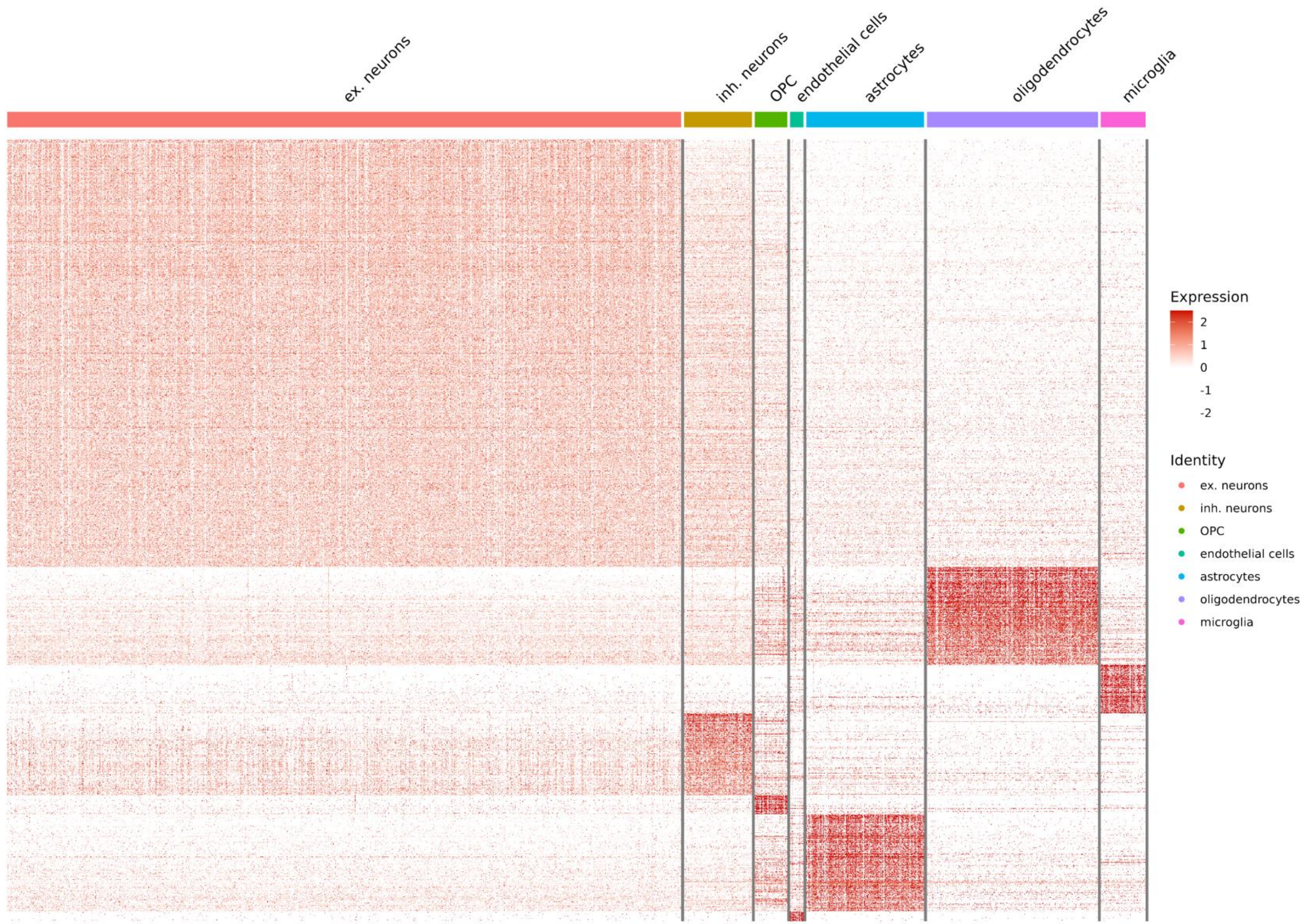

Supplementary Figure 5

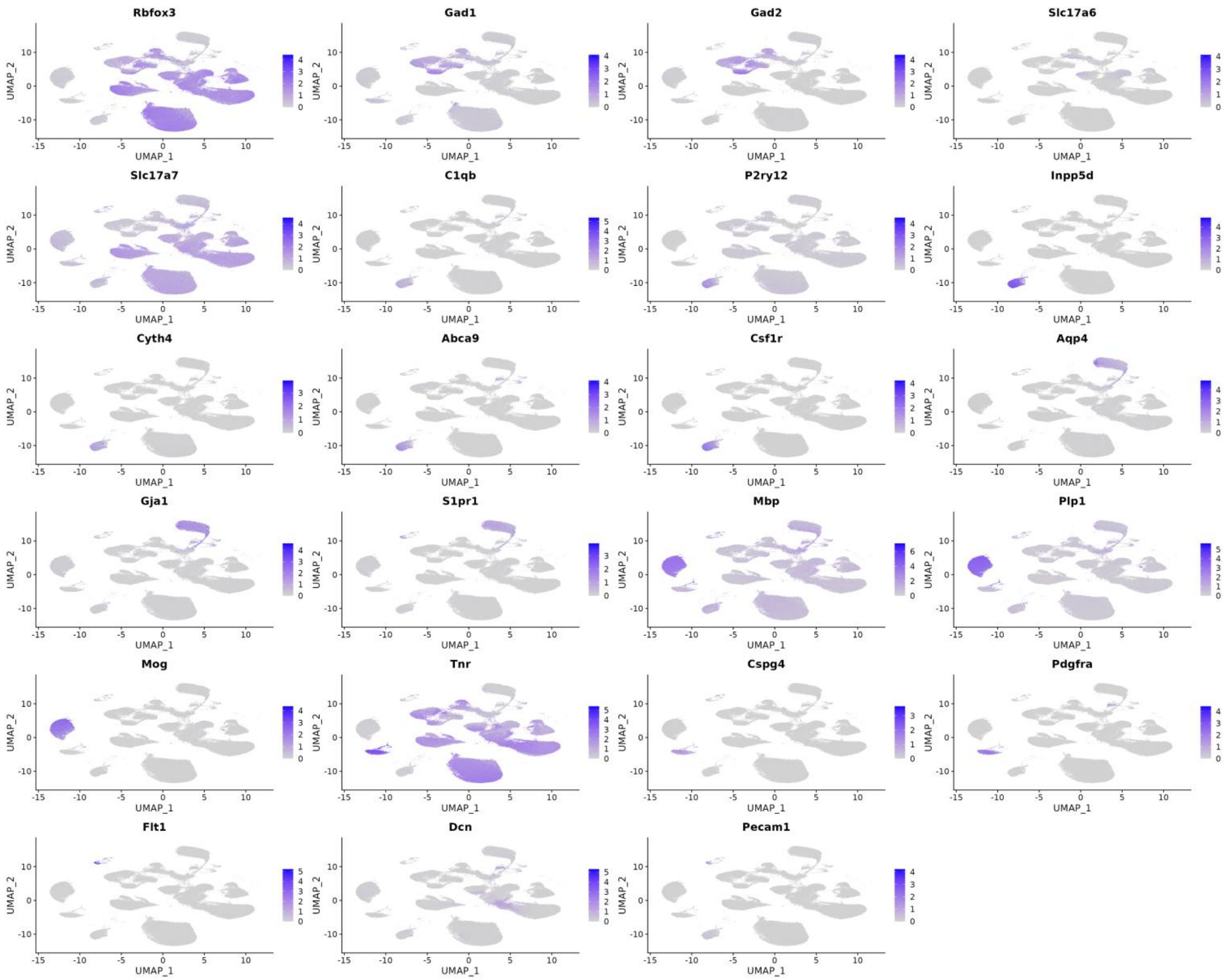

Supplementary Figure 6

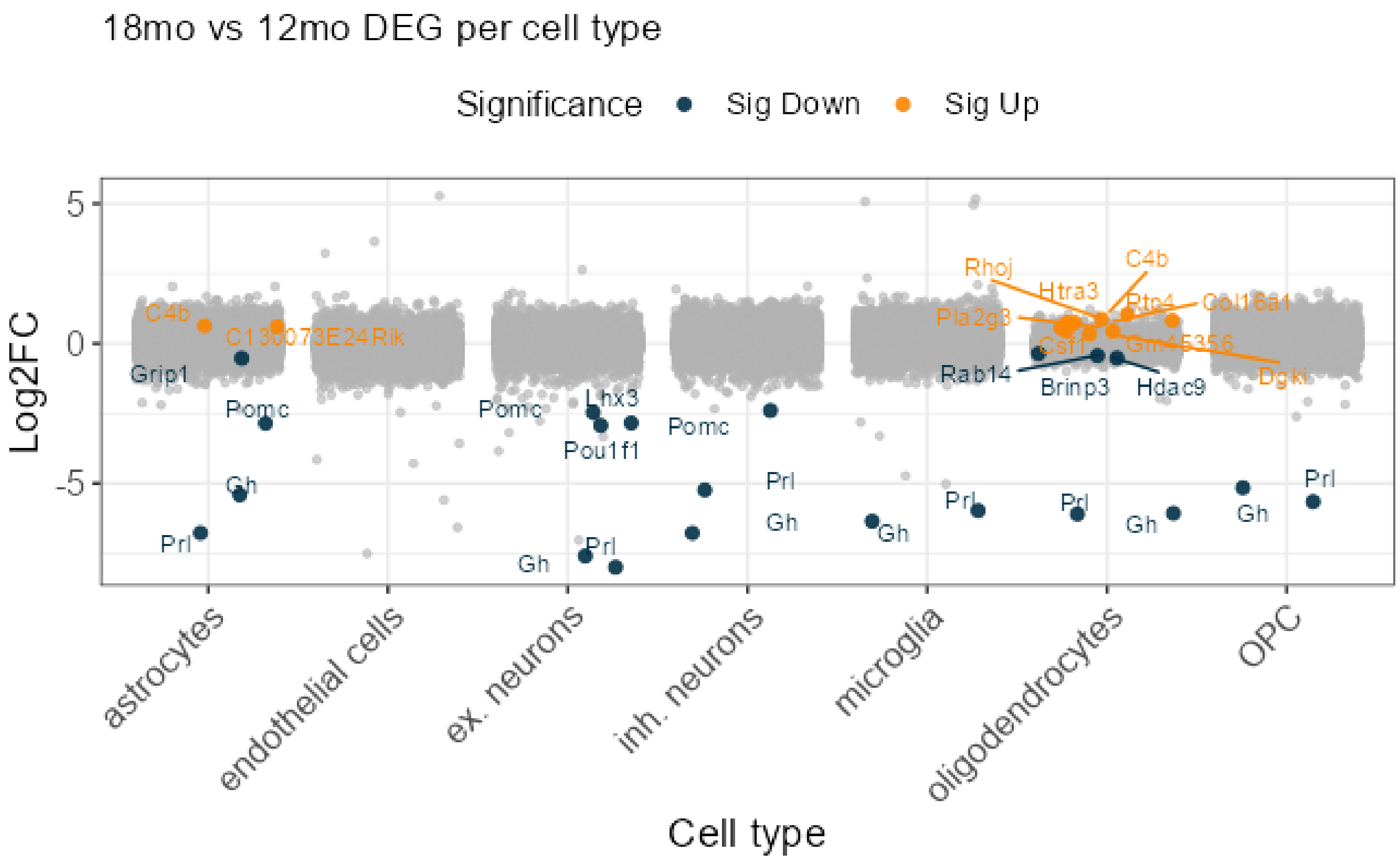

Supplementary Figure 7

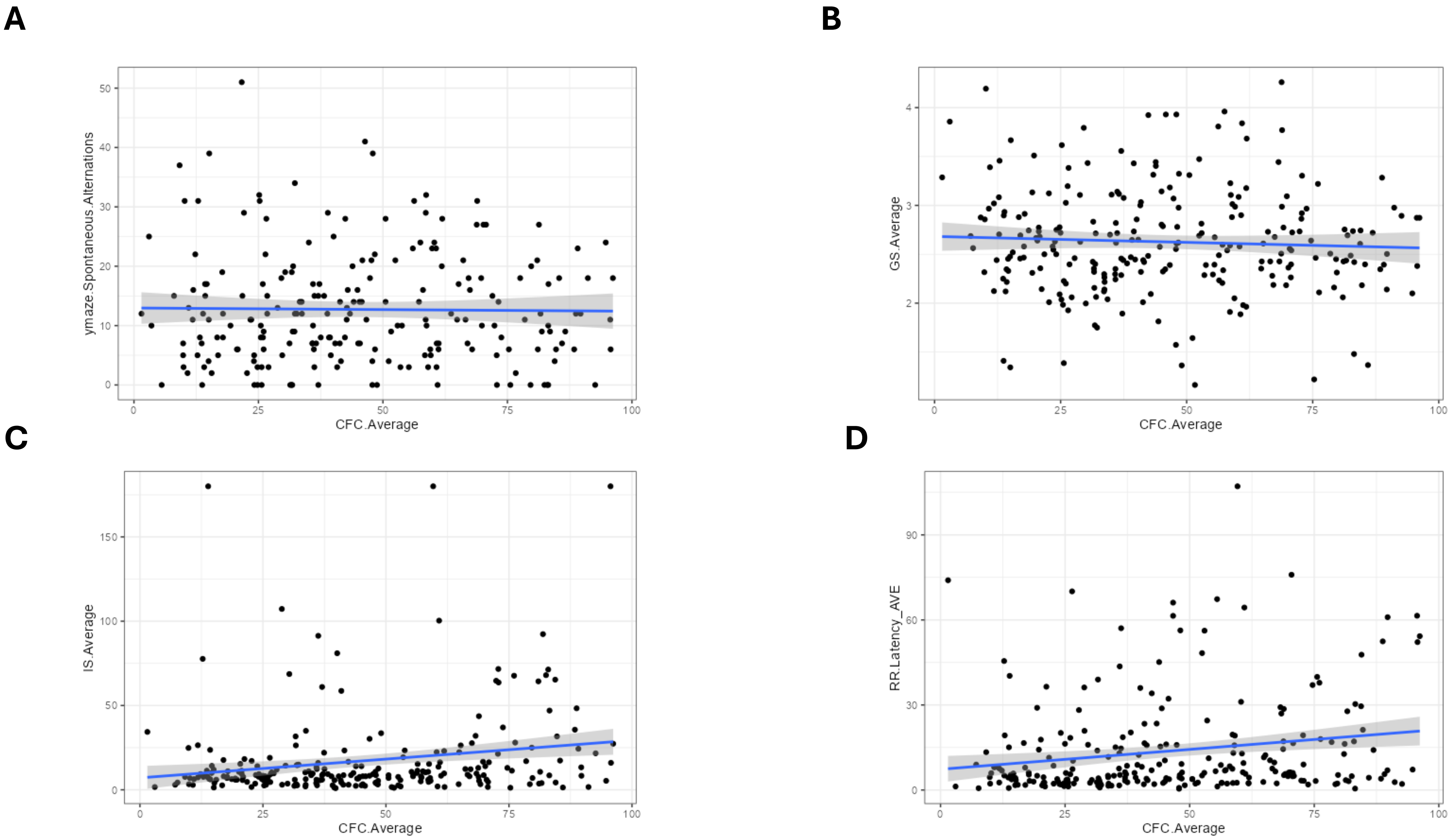

Supplementary Figure 8

A

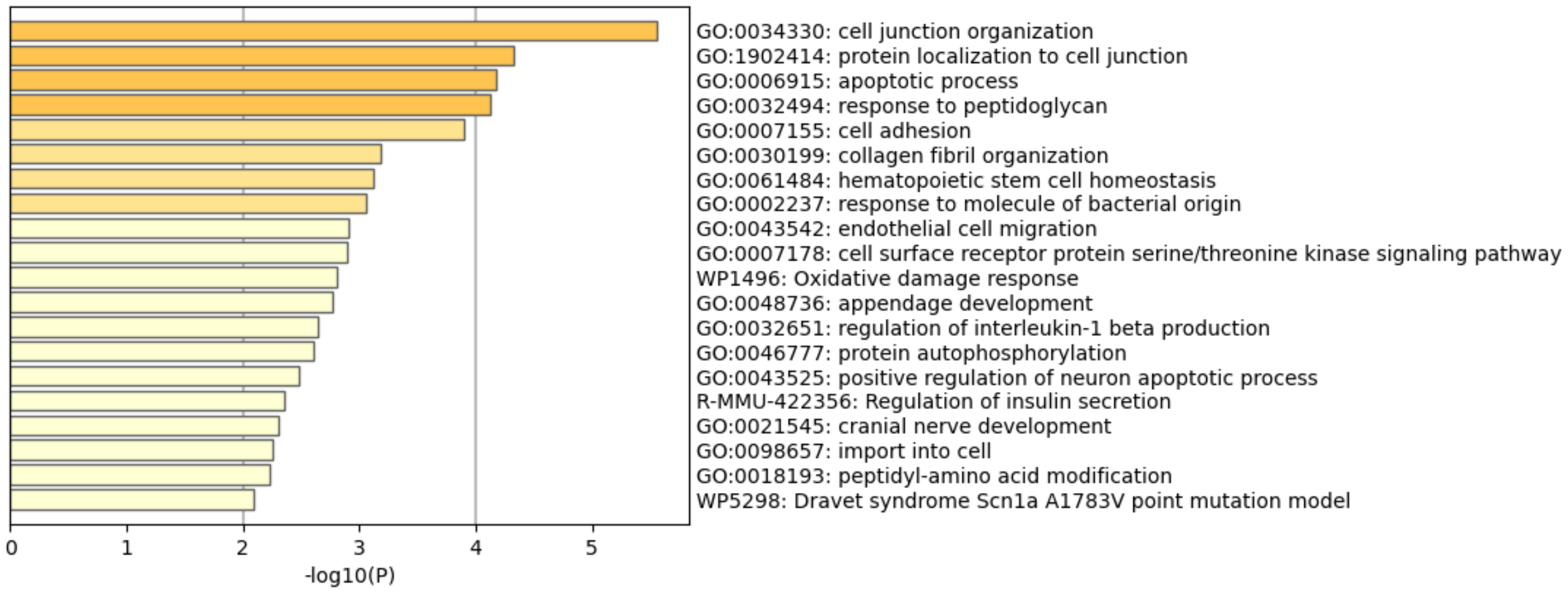

B

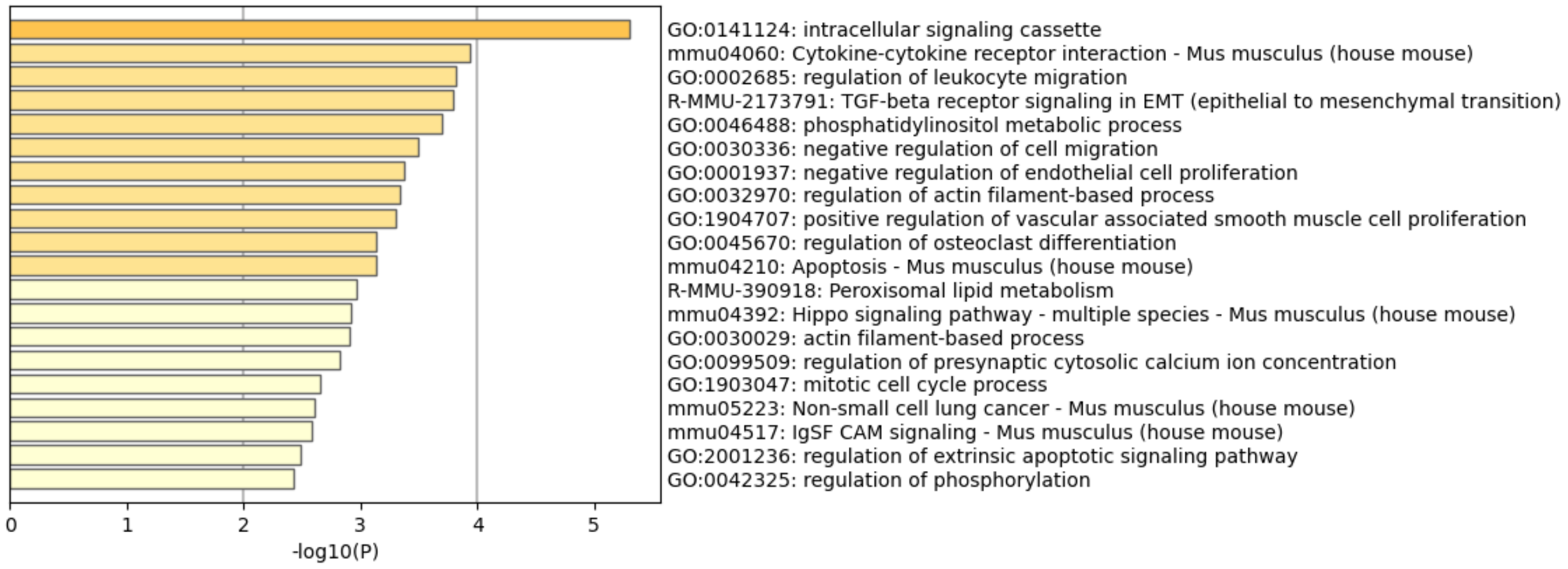

Supplementary Figure 9

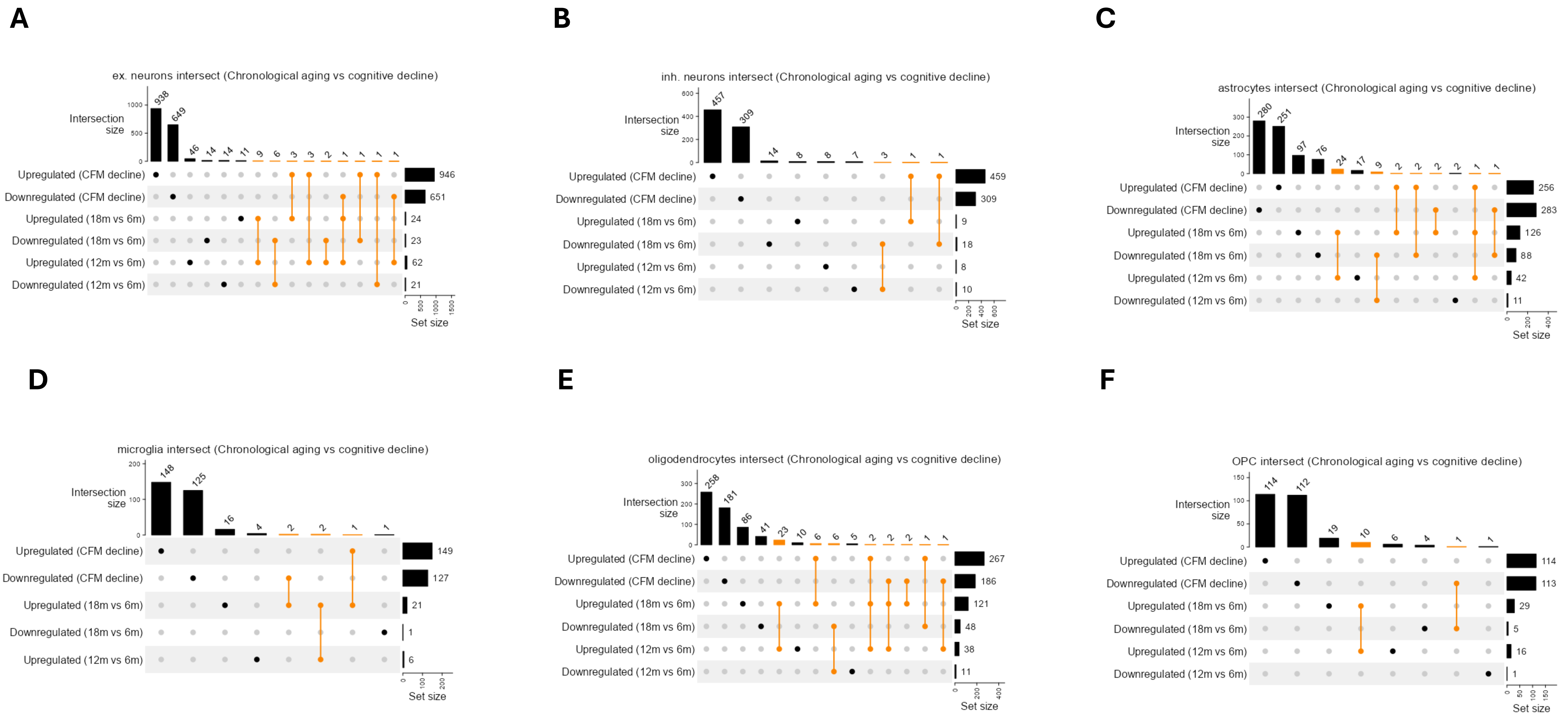

Supplementary Figure 10

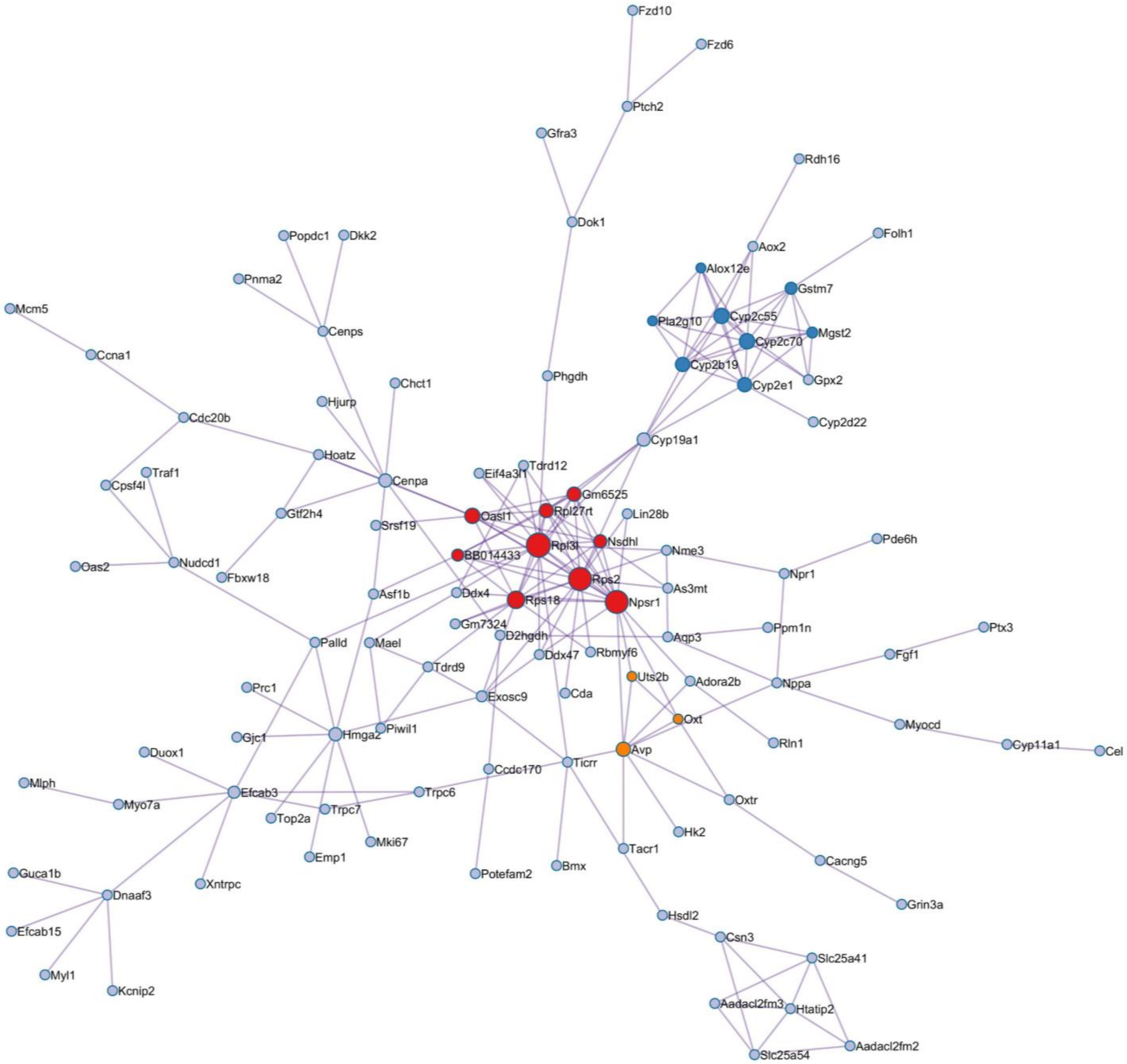

Supplementary Figure 11

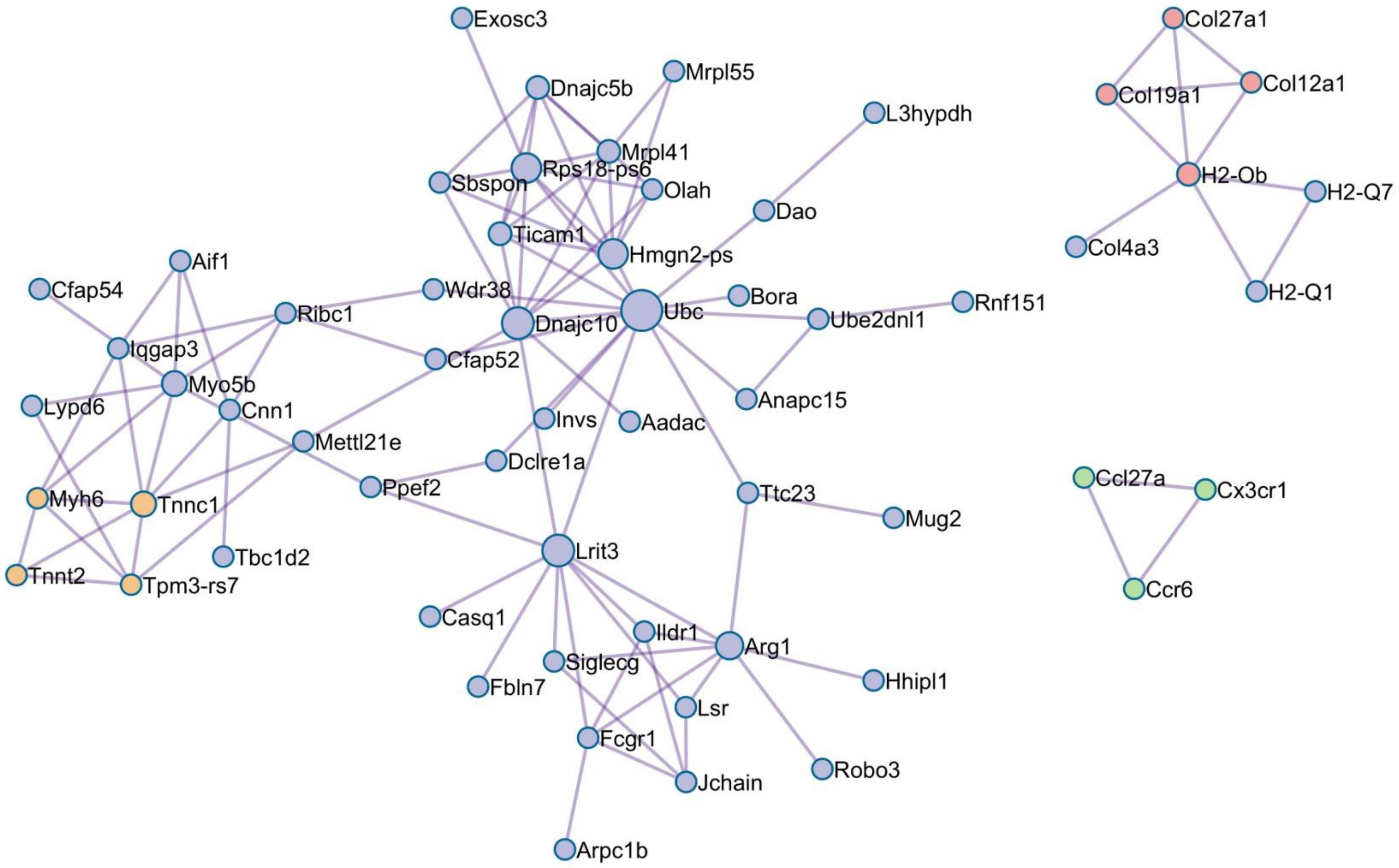

Supplementary Figure 12

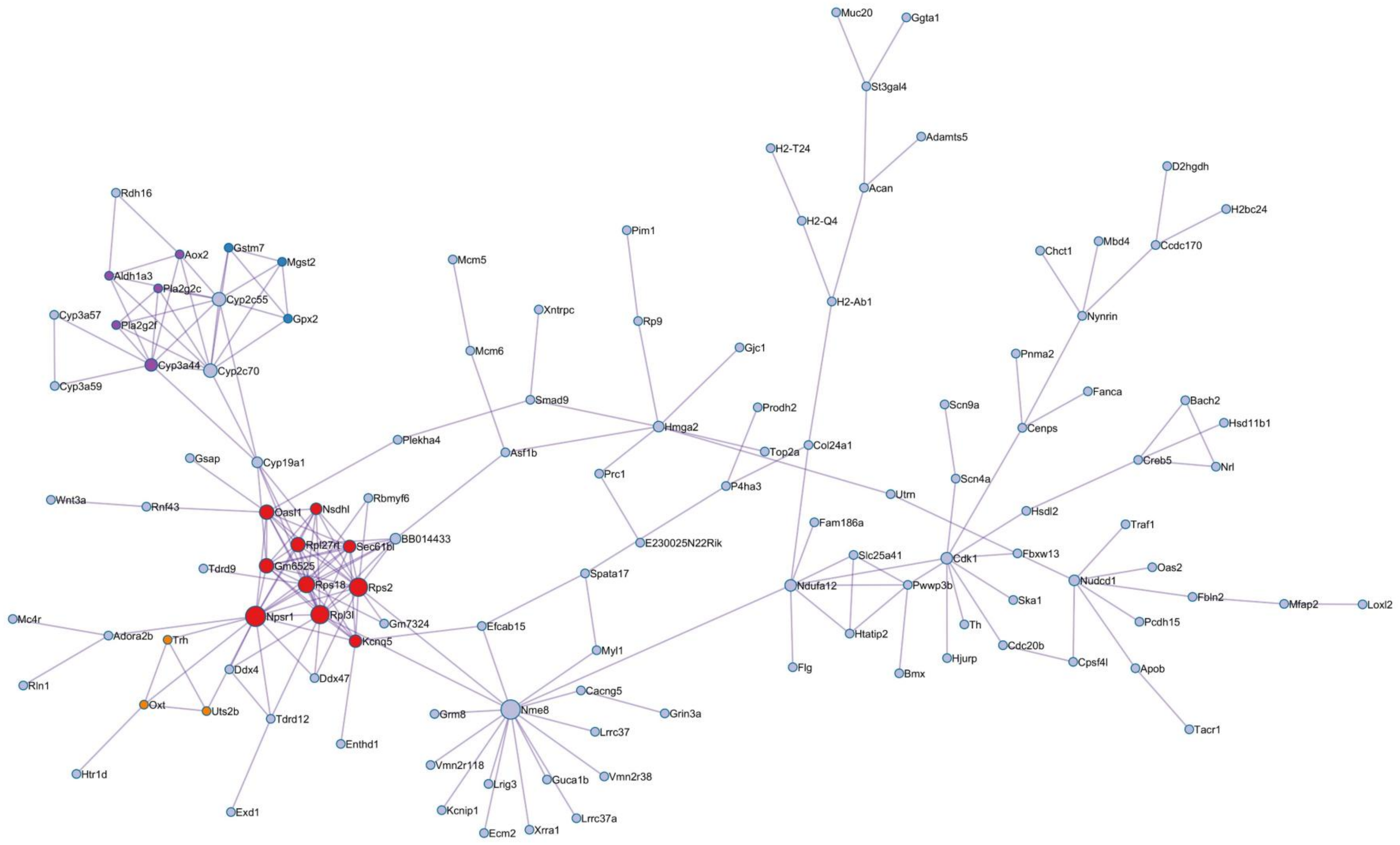

Supplementary Figure 13

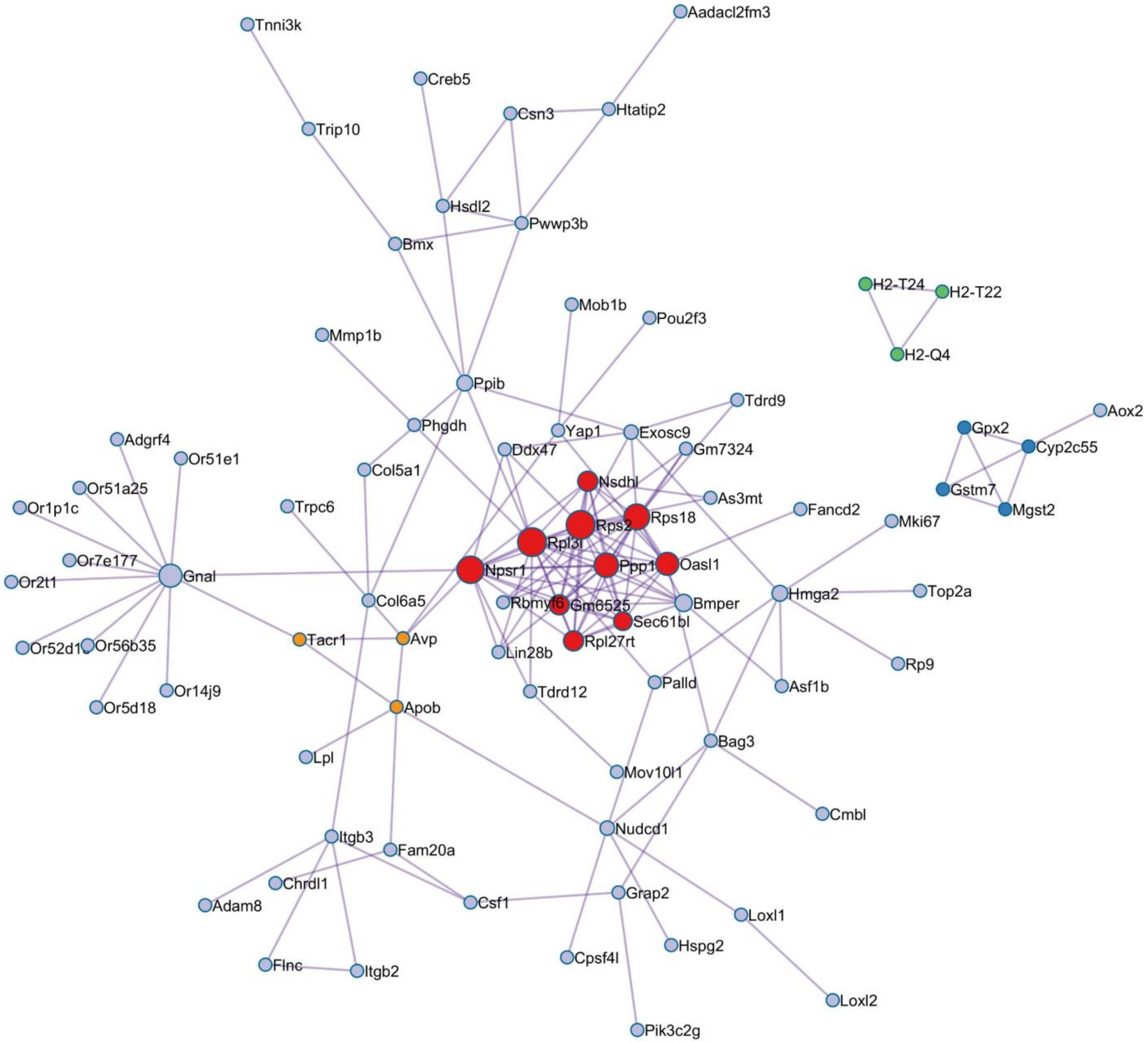

Supplementary Figure 14

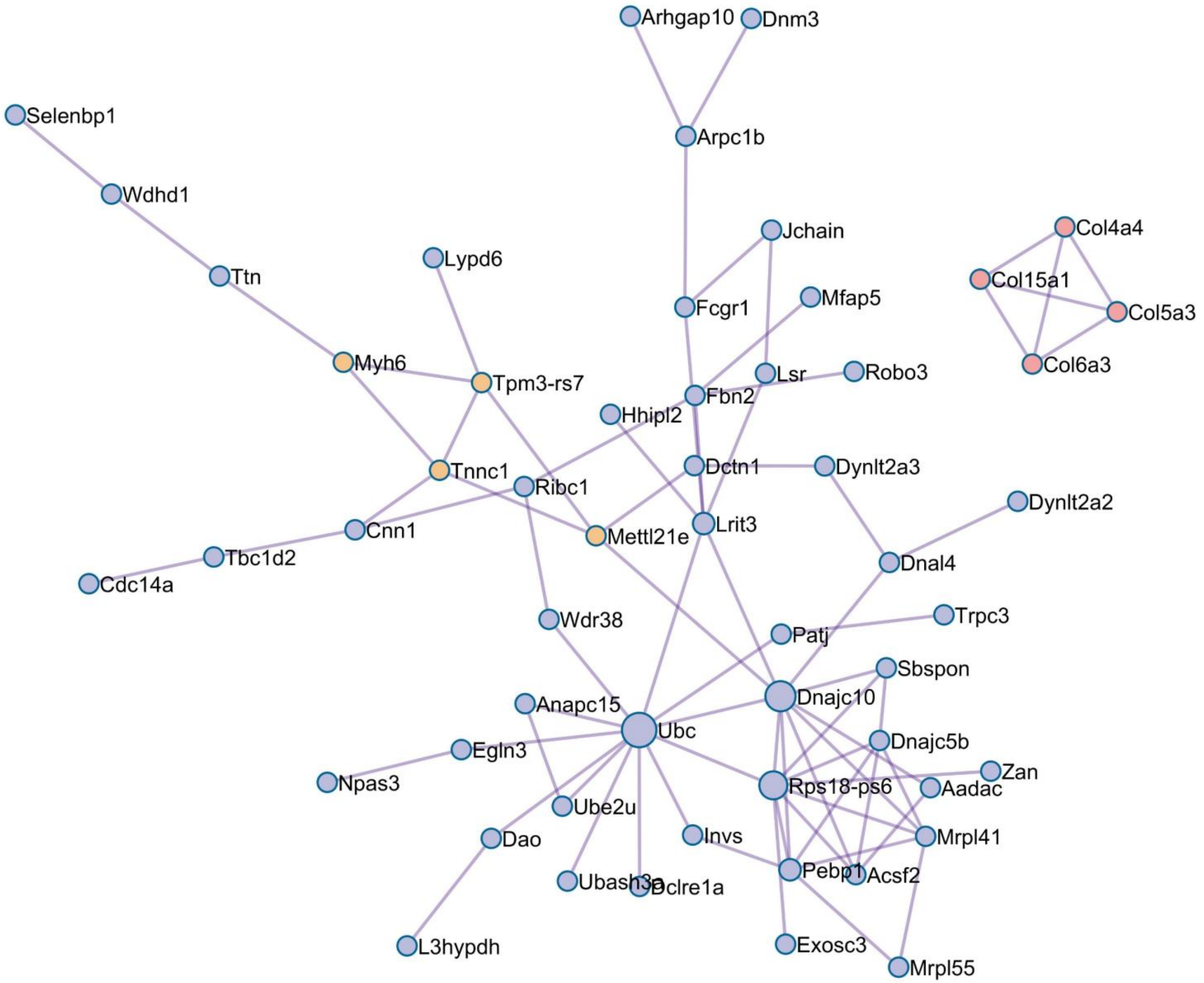

Supplementary Figure 15

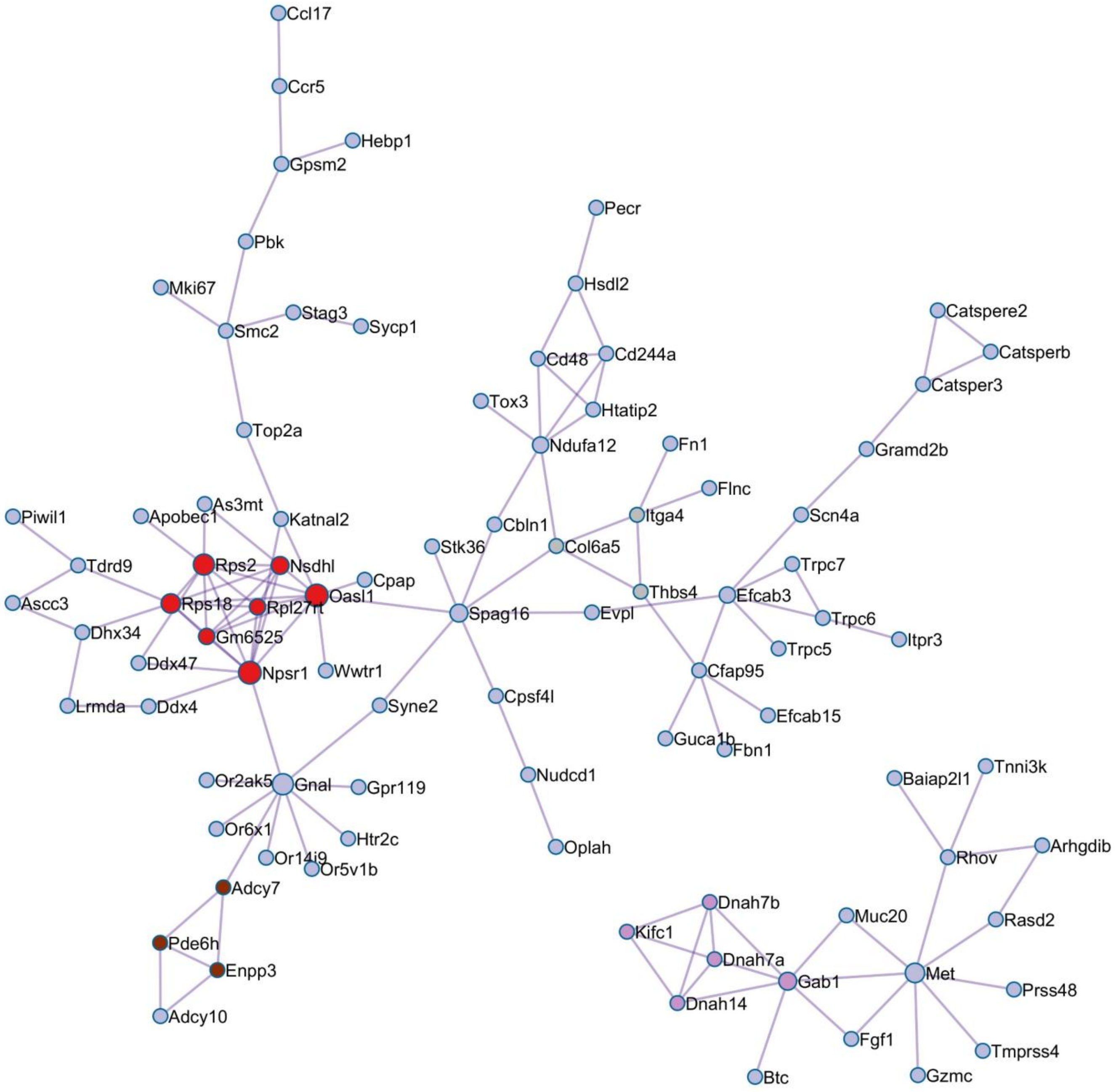

Supplementary Figure 16

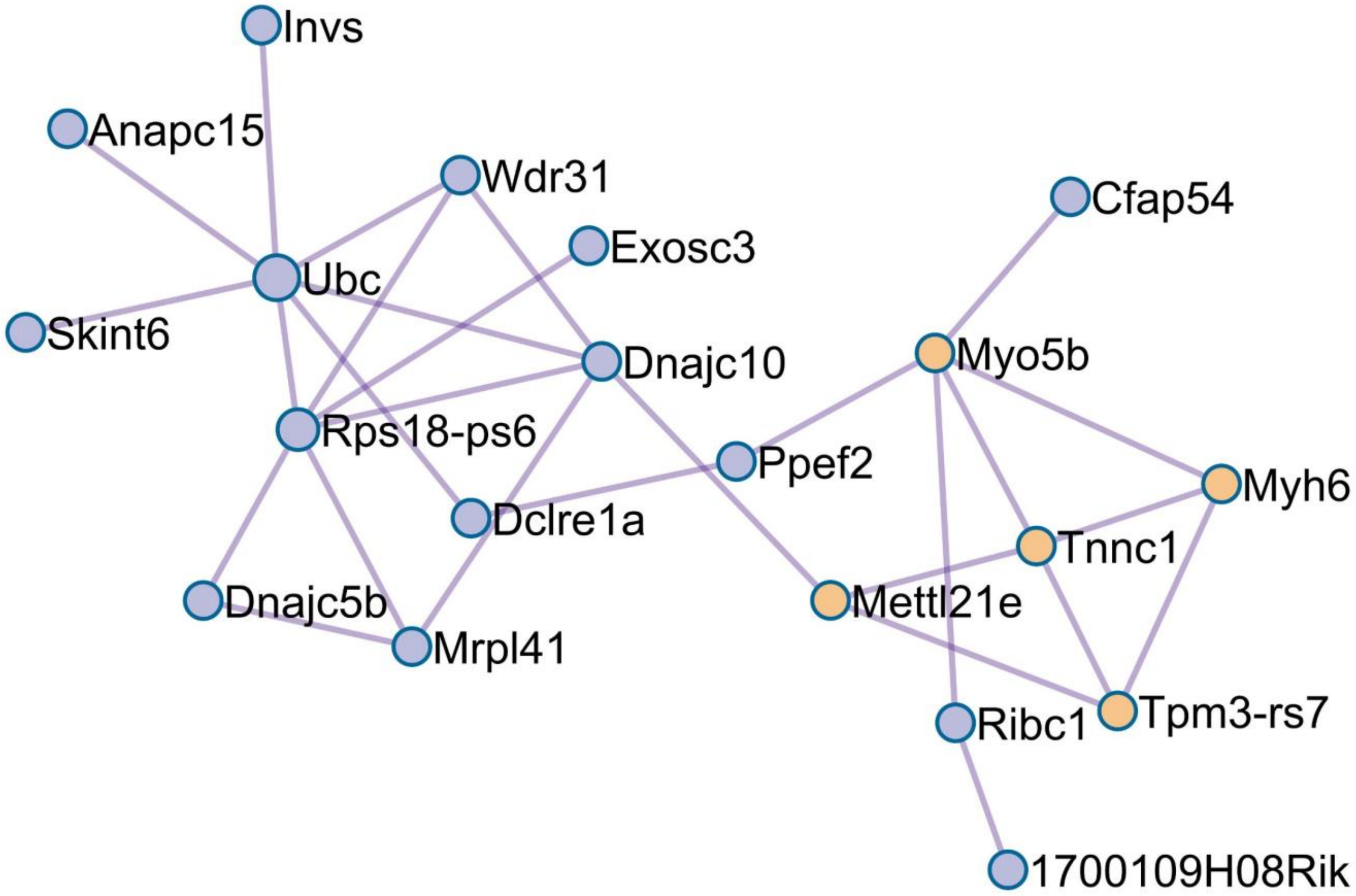

Supplementary Figure 17

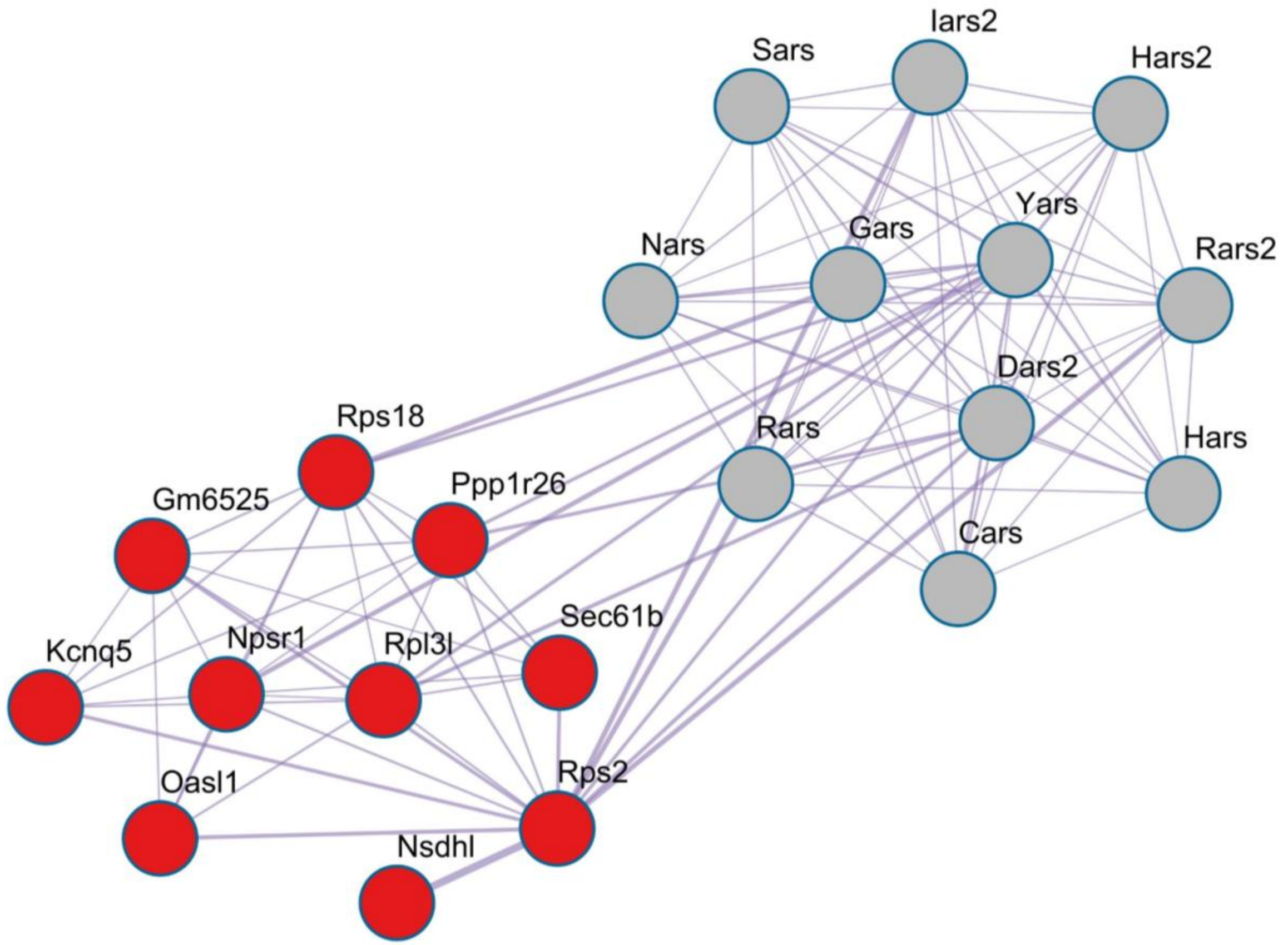

Supplement: Supplementary file 1 [file ALZ-22-e71825-s001.pdf]
